# Nutraceutical Chewing Candy Formulations Based on Acetic, Alcoholic, and Lactofermented Apple Juice Products

**DOI:** 10.3390/foods10102329

**Published:** 2021-09-30

**Authors:** Elena Bartkiene, Egle Zokaityte, Paulina Zavistanaviciute, Ernestas Mockus, Darius Cernauskas, Modestas Ruzauskas, Ernesta Tolpeznikaite, Raquel P. F. Guiné

**Affiliations:** 1 Department of Food Safety and Quality, Faculty of Veterinary Medicine, Lithuanian University of Health Sciences, Tilzes Str. 18, LT-47181 Kaunas, Lithuania; elena.bartkiene@lsmuni.lt (E.B.); paulina.zavistanaviciute@lsmuni.lt (P.Z.); 2 Institute of Animal Rearing Technologies, Faculty of Animal Sciences, Lithuanian University of Health Sciences, Tilzes Str. 18, LT-47181 Kaunas, Lithuania; ernestas.mockus@lsmuni.lt (E.M.); darius.cernauskas@lsmuni.lt (D.C.); ernesta.tolpeznikaite@lsmuni.lt (E.T.); 3 Department of Anatomy and Physiology, Faculty of Veterinary Medicine, Lithuanian University of Health Sciences, Tilzes Str. 18, LT-47181 Kaunas, Lithuania; modestas.ruzauskas@lsmuni.lt; 4 Institute of Microbiology and Virology, Faculty of Veterinary Medicine, Lithuanian University of Health Sciences, Tilzes Str. 18, LT-47181 Kaunas, Lithuania; 5 CERNAS Research Centre, Polytechnic Institute of Viseu, 3504-510 Viseu, Portugal; raquelguine@esav.ipv.pt

**Keywords:** nutraceutical chewing candy, apple juice, fermentation, lactic acid bacteria, antimicrobial properties, volatile compounds

## Abstract

The aim of this study was to develop nutraceutical chewing candy (NCC) formulations based on acetic, alcoholic, and lactofermented apple juice (AJ) products. In addition, different texture-forming (gelatin, pectin) and sweetening (stevia, xylitol) agents were tested. To implement the aim of this study, combinations based on AJ, prepared from fresh and frozen apples, apple cider (C) samples (No.1, No.2, No.3, and No.4), and apple vinegar (V) were used. First, the most appropriate combination was selected by evaluating overall acceptability (OA) and emotions induced for consumers (EIC). In addition, the volatile compound (VC) profile, and physicochemical and antimicrobial characteristics of the developed combinations were analyzed. For AJ fermentation, lactic acid bacteria (LAB) strains possessing antimicrobial properties (LUHS122—*L. plantarum* and LUHS210—*L. casei*) were used. AJ prepared from frozen apples had 11.1% higher OA and 45.9%, 50.4%, and 33.3% higher fructose, glucose, and saccharose concentrations, respectively. All the tested C samples inhibited *Bacillus subtilis* and had an average OA of 6.6 points. Very strong positive correlations were found between AJ and C OA and the emotion ‘happy’; comparing lactofermented AJ, the highest OA was obtained for AJ fermented for 48 h with LUHS122, and a moderate positive correlation was found between AJ OA and the emotion ‘happy’ (r = 0.7617). This sample also showed the highest viable LAB count (7.59 log_10_ CFU mL^−1^) and the broadest spectrum of pathogen inhibition (inhibited 6 out of 10 tested pathogens). Further, acetic, alcoholic, and lactofermented AJ product combinations were tested. For the preparation of NCC, the combination consisting of 50 mL of AJ fermented with LUHS122 for 48 h + 50 mL C-No.3 + 2 mL V was selected because it showed the highest OA, induced a high intensity of the emotion ‘happy’ for the judges, and inhibited 8 out of 10 tested pathogens. Finally, the OA of the prepared NCC was, on average, 9.03 points. The combination of acetic, alcoholic, and lactofermented AJ products leads to the formation of a specific VC profile and increases the OA and antimicrobial activity of the products which could be successfully applied in the food and nutraceutical industries.

## 1. Introduction

According to the Food and Agriculture Organization (FAO) of the United Nations, apple is one of the most important and popular fruit species in the World [1,2,3], because of its good sensory properties and health benefits [4]. The main apple products are juice, cider, vinegar, and fermented apple juice [5], as well as dehydrated [6,7,8,9], canned [1,10], and purées [11,12].

Nowadays, fermented products, including apple juice, have become very popular because of their health benefits and good sensory properties. Fermentation increases the bioavailability of bioactive substances (polyphenols, etc.) and the nutritional value of the fermentable substrate [13,14,15]. Consumers are looking for non-dairy substrates (cereals, vegetables, fruits, etc.) fermented by functional microorganisms (probiotics, possessing antimicrobial properties, etc.) [5]. Non-dairy substrates have more bioactive compounds, are cheaper, and can be used by the lactose-intolerant population [5]. Fermented beverages possess many desirable properties: stronger antioxidant characteristics, gastrointestinal health improvement [16], and possibly possessing antimicrobial properties [17]. These characteristics may be due to technological microorganisms and their metabolites in fermented juice: organic acids, vitamins, phenolic compounds, etc. [18]. However, the characteristics of fermented products depend on the microorganism(s) used, the fermentable substrate, and the fermentation conditions [19,20,21].

Taking into consideration that most (bio)converted (fermented) apple juice products possess desirable health benefits for consumers, in this study, we hypothesized that the combination of acetic, alcoholic, and lactofermented apple juice (AJ) products could have increased value, in comparison with the separate products, because of the greater variety of compounds formed (volatile, antimicrobial, etc.), and could be valuable ingredients for developing nutraceutical chewing candy (NCC) formulations. To select the best combinations, in addition to the standard sensory analysis method, an emotion intensity scanning technique (FaceReader software) was applied in this study, as we hypothesized that the implicit emotional responses revealed through facial expressions could indicate the interaction of consumers with products in a more sensitive manner, because emotions have a significant role in the comprehension of food preferences and consumer acceptability.

The aim of this study was to develop NCC formulations based on acetic, alcoholic, and lactofermented AJ products. In addition, different texture-forming (gelatin, pectin) and sweetening (stevia, xylitol) agents were tested. First, the most appropriate combination was selected by evaluating the overall acceptability and emotions induced for consumers. The volatile compound profile, and physicochemical and antimicrobial characteristics of the developed combinations were also analyzed. For AJ fermentation, lactic acid bacteria (LAB) strains possessing antimicrobial properties (LUHS122—*L. plantarum* and LUHS210—*L. casei*) were used.

## 2. Materials and Methods

### 2.1. Principle Scheme of the Experiment and Materials Used for Preparation of Nutraceutical Chewing Candies

**Figure 1:**
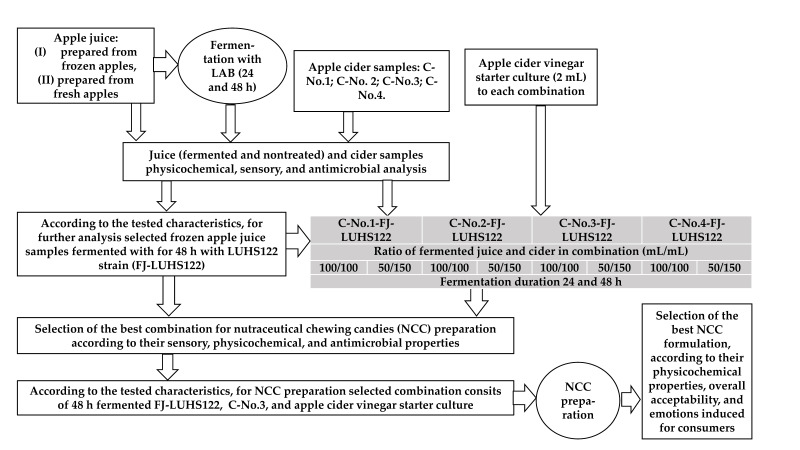
Principal scheme of the experiment.

The principal scheme of the experiment is shown in Figure 1. In the first stage of the experiment, different AJ products were tested (two types of ‘*Ligol*’ variety AJ: (I) prepared from frozen apples (before juice preparation, apples were frozen at −18 °C for 24 h), (II) prepared from fresh apples, as well as four apple cider (C) samples, prepared at the small industrial scale agricultural company ‘Auseklis’ (Ardiskis, Lithuania) using the same technology and the same variety of apples (‘*Ligol*’) (C-No.1 (1 December 2019), C-No.2 (4 December 2020), C-No.3 (28 October 2020), and C-No.4 (9 December 2020)). Apple cider vinegar (V) starter culture was obtained from the same company and used for the development of products combining acetic, alcoholic, and lactofermented apple products.

During the first stage of the experiment, AJ and C samples were analyzed, and their pH, dry matter, overall acceptability, emotions induced for consumers, and antimicrobial properties were tested. Then, both of the tested AJ samples were fermented (24 and 48 h) with the antimicrobial LAB strains *L. plantarum* LUHS122 and *L. casei* LUHS210 [22] (in total, eight samples were obtained).

The *L. plantarum* LUHS122 and *L. casei* LUHS210 strains were incubated separately and multiplied in De Man, Rogosa, and Sharpe (MRS) broth culture medium (Biolife, Milan, Italy) at 30 °C under anaerobic conditions. AJ (100 mL) was inoculated with 1 mL of LAB multiplied in MRS (average cell concentration 9.0 log_10_ CFU mL^−1^) followed by anaerobic fermentation in a modified carbon dioxide atmosphere in a chamber incubator (Memmert GmbH + Co. KG, Schwabach, Germany) for 24 and 48 h at 30 °C. Fermented AJ samples were selected according to their overall acceptability, emotions induced for consumers, antimicrobial properties, and viable LAB count. In addition, the volatile compound (VC) profile, sugar concentration, and color coordinates of the fermented and non-fermented AJ were analyzed.

In parallel to the experiment with AJ, C samples were tested. To select the most appropriate C for NCC formulation, overall acceptability, emotions induced for consumers, and antimicrobial properties were analyzed.

Then, combinations of the selected AJ, C, and V were tested. Compounds were mixed at different ratios (Figure 1), and fermentation of the combinations for different durations was tested (24 and 48 h). The combinations were analyzed and the most appropriate one was selected according to overall acceptability, emotions induced for consumers, and antimicrobial properties. In addition, the VC profile, ethanol and sugar concentrations, pH, and color coordinates of the developed combinations were tested.

During the second stage of the experiment, the selected combination was used to prepare NCC. For this purpose, several formulations were tested (Table 1), using two texture-forming agents (gelatin and pectin) and two sweetening agents (stevia and xylitol). To increase the intensity of the sour taste of the NCC, fresh pressed lemon juice was included in one of the formulations. To select the best NCC recipe, overall acceptability, emotions induced for consumers, texture, and color coordinates were analyzed.

The LAB strains used in this study were previously isolated from spontaneously fermented cereal and showed a broad spectrum of antimicrobial properties [22].

All the apple products tested (juice, cider, apple cider vinegar starter culture) were obtained from the small industrial scale agricultural company ‘Auseklis’ (Ardiskis, Lithuania), and stored at −18 °C before use.

Gelatin (Klingai, Kaunas, Lithuania) and pectin were obtained from Sosa (Rome, Italy). Xylitol was obtained from Natur Hurtig (Nuremberg, Germany), and stevia was purchased from SteviaBalt (Jelgava, Latvia).

### 2.2. Preparation of Nutraceutical Chewing Candies

For preparation of NCC with gelatin, first, gelatin powder was soaked in water (16 g gelatin and 25 mL water) for 30 min, and then melted at 80 ± 2 °C and cooled to 30 ± 2 °C, then other ingredients were added and mixed.

For preparation of NCC with pectin, first, pectin powder was soaked in water (6 g pectin and 25 mL water) for 30 min, and then melted by heating for 5 min and cooled to 30 ± 2 °C, then other ingredients were added and mixed.

The NCC mass was poured into a mold, and NCC were dried at 22–24 °C for 24 h to get a hard gel form.

### 2.3. Evaluation of Overall Acceptability and Emotions Induced for Consumers by the Tested Apple Juice-Based Products

The overall acceptability of the developed combinations and prepared NCC was established by 35 judges, according to International Standards Organization method 8586-1 [23], using a 10-point scale ranging from 0 (‘dislike extremely’) to 10 (‘like extremely’). Samples were also tested by applying FaceReader 8.0 software (Noldus Information Technology, Wageningen, The Netherlands), scaling eight emotion patterns (neutral, happy, sad, angry, surprised, scared, disgusted, contempt) according to the procedure described by Bartkiene et al. [24].

### 2.4. Analysis of Colour Characteristics, Texture, pH, and Dry Matter

The color coordinates (L*, a*, b*) were assessed using a CIELAB system (Chromameter CR-400, Konica Minolta, Tokyo, Japan).

Texture was evaluated using a Brookfield CT-3 Texture Analyser (Ametek GmbH, B.U. Brookfield, Hadamar-Steinbach, Germany).

The pH was measured using a pH electrode (PP-15; Sartorius, Goettingen, Germany). The dry matter of samples was evaluated with a saccharometer (Merck KGaA, Darmstadt, Germany).

### 2.5. Analysis of the Fructose, Glucose, Sucrose, Maltose, and Ethanol Concentrations in Apple Products

To determine the sugar concentration, 2–3 mL of sample was diluted with ~70 mL of distilled/deionized water, heated to 60 °C in a water bath for 15 min, clarified with 2.5 mL of Carrez I (85 mM K_4_[Fe(CN)_6_] × 3H_2_O) (Sigma-Aldrich, Darmstadt, Germany) and 2.5 mL of Carrez II (250 mM ZnSO_4_ × 7H_2_O) (Sigma-Aldrich, Darmstadt, Germany) solutions, and made up to 100 mL with distilled/deionized water. After 15 min, the samples were filtered through a filter paper and a 0.22 μm nylon syringe filter (Sigma-Aldrich, Darmstadt, Germany) before analysis. A standard solution of a sugar’s mixture was prepared by dissolving 0.2 g each of fructose (Sigma-Aldrich, Darmstadt, Germany), glucose (Sigma-Aldrich, Darmstadt, Germany), sucrose (Sigma-Aldrich, Darmstadt, Germany), and maltose (Sigma-Aldrich, Darmstadt, Germany) in 100 mL of distilled/deionized water. A 2 mg mL^−1^ standard solution of sugar mixture was prepared following dilution with distilled/deionized water. Chromatographic conditions were as follows: the eluent was a mixture of 75 parts by volume of acetonitrile and 25 parts by volume of water; the flow rate was 1.2 mL min^−1^; 20 μL was injected. A YMC-Pack Polyamine II column (250 × 4.6 mm, 5 μm; YMC Co., Ltd., Kyoto, Japan) was used. The column temperature was set at 28 °C. Detection was performed using an ELSDLTII Evaporative Light Scattering Detector (Shimadzu Corp., Tokyo, Japan).

Ethanol concentrations were determined enzymatically using a Megazyme Ethanol Assay Kit (Megazyme, Bray, Ireland). The quantification of ethanol requires two enzyme reactions; in the first reaction catalyzed by alcohol dehydrogenase (ADH), ethanol is oxidized to acetaldehyde by nicotinamide-adenine dinucleotide (NAD+). However, since the equilibrium of reaction lies in favor of ethanol and NAD+, a further reaction is required to ‘trap’ the products. This is achieved by the quantitative oxidation of acetaldehyde to acetic acid in the presence of aldehyde dehydrogenase (Al-DH) and NAD+. The amount of NADH formed in this reaction pathway is stoichiometric with twice the amount of ethanol. It is the NADH which is measured by the increase in absorbance at 340 nm.

### 2.6. Determination of Viable Lactic Acid Bacteria Count

To evaluate the viable LAB count, 10 g of sample was homogenized with 90 mL of saline (9 g L^−1^ NaCl solution). Serial dilutions of 10^−4^ to 10^−8^ with saline were used for sample preparation. Sterile MRS agar (5 mm thick; CM0361, Oxoid, Hampshire, UK) was used for bacterial growth on Petri dishes. The dishes were separately seeded with the sample suspension using surface sowing and were incubated under anaerobic conditions at 30 °C for 72 h. All results were expressed in log_10_ CFU mL^−1^ (colony-forming units per mL of sample) as the mean and standard deviation of three determinations.

### 2.7. Evaluation of Antimicrobial Properties

The antimicrobial activity of AJ and C samples, as well as their combinations, were assessed for their antimicrobial activity against a variety of pathogenic and opportunistic bacterial strains (*Escherichia coli*, *Klebsiella pneumoniae*, *Salmonella enterica*, *Cronobacter sakazakii*, *Acinetobacter baumannii*, *Pseudomonas aeruginosa*, *Staphylococcus aureus*, *S. haemolyticus*, *Bacillus subtilis*, and *Streptococcus mutans*) by using the agar well diffusion method. For this purpose, suspensions of 0.5 McFarland standard of each pathogenic bacterial strain were inoculated onto the surface of cooled Mueller–Hinton agar (Oxoid, Basingstoke, UK) using sterile cotton swabs. Wells 6 mm in diameter were punched in the agar and filled with 50 µL of the tested sample. The antimicrobial activity against the tested bacteria was established by measuring the inhibition zone diameters (mm). The experiments were repeated three times, and the average diameter of the inhibition zones was calculated.

### 2.8. Determination of Volatile Compound Profile

The VC of juice and cider samples were analyzed by gas chromatography-mass spectrometry (GC-MS). Solid phase microextraction (SPME) device with Stableflex (TM) fiber coated with 50 µm PDMS-DVB-Carboxen™ layer (Supelco, Bellefonte, PA, USA) was used for analysis. For headspace extraction 2 mL of cider or juice sample were added to the 20 mL extraction vial containing 3 g of sodium chloride and diluted with 8 mL of deionized water. The extraction vial contents were mixed, sealed with polytetrafluoroethylene septa and thermostated at 60 °C for 15 min before exposing the fiber in the headspace. The fiber was exposed to the headspace of the vial for 10 min and desorbed in the injector liner for 2 min (splitless injection mode). Prepared samples were analyzed with GCMS-QP2010 (Shimadzu, Kyoto, Japan) gas chromatograph and a mass spectrometer. The following method conditions were used for analysis: injector temperature 250 °C, ion source temperature 220 °C, interface temperature 280 °C. Helium (99.999% detector purity, AGA, Vilnius, Lithuania) was used as carrier gas at 0.97 mL/min flow rate. Rxi^®^-5MS column (0.25 mm ID, 0.25 μm film thickness, 30 m length (Restek, Bellefonte, PA USA)) capillary column was used for analysis. Temperature gradient was programmed from start at 35 °C (5 min hold) to 200 °C (10 °C/min) up to 280 °C (25 °C/min) (5 min hold). The VC were identified according to the mass spectra libraries (NIST11, NIST11S, FFNSC2).

### 2.9. Statistical Analysis

The results were expressed as the mean ± standard deviation (SD). Preparation of NCC was performed twice; all analyses were performed in triplicate. Results were analyzed using the statistical package SPSS for Windows V15.0 (SPSS Inc., Chicago, IL, USA, 2007). One sample T-Test was used to compare results, also, a linear Pearson’s correlation was used to quantify the strength of the relationship between the variables. Results were recognized as statistically significant at *p* ≤ 0.05.

## 3. Results and Discussion

### 3.1. Characterization and Selection of Apple Juice and Cider Samples

The overall acceptability and emotions induced for consumers by AJ and C samples are shown in Table 2. In comparing AJ samples, it was found that AJ prepared from frozen apples had 11.1% higher overall acceptability than AJ prepared from fresh apples.

The overall acceptability of all the C samples was, on average, 6.6 points. A very strong positive correlation was found between the overall acceptability of AJ and C and the emotion ‘happy’ (r = 0.9173), and moderate negative correlations were established between the overall acceptability of AJ and C samples and the emotions ‘angry’ and ‘surprised’ (r = −0.6360 and r = −0.5988, respectively).

The physicochemical parameters of the samples (Table 2) showed correlations with the overall acceptability, and increasing pH and lightness (L*) reduced the overall acceptability of the samples (r = −0.7032 and r = −0.8401, respectively). However, increasing dry matter (DM), redness (a*), and yellowness (b*) increased overall acceptability (r = −0.8394, r = −0.7836, and r = −0.8776, respectively).

Saccharide and ethanol concentrations in non-fermented AJ and C samples are shown in Table 3. Comparing AJ samples, juice prepared from frozen apples had 45.9%, 50.4%, and 33.3% higher fructose, glucose, and saccharose concentrations, respectively, than juice prepared from fresh apples. No ethanol was determined in AJ; in C samples, the ethanol concentration was, on average, 2.62 g 100 g^−1^. Fructose was found in C samples No.3 and No.4 (0.781 and 0.490 g 100 g^−1^, respectively).

The antimicrobial properties of AJ and C are shown in Table 4.

It was found that AJ (prepared from both frozen and fresh apples) and C sample No.1 inhibited *Bacillus subtilis* (diameter of inhibition zone 11.2, 8.34, and 8.10 mm, respectively). However, AJ and C samples did not show antimicrobial properties against the other pathogens tested.

According to the results obtained, frozen AJ and all the tested C samples were used for the development of combinations for preparation of NCC.

### 3.2. Characteristics of the Fermented Apple Juice

During the second stage of the experiment, AJ was fermented with antimicrobial LAB strains (LUHS122—*L. plantarum*; LUHS210—*L. casei*), and different durations of fermentation were tested (24 and 48 h).

The overall acceptability and emotions induced for consumers by the fermented AJ samples are shown in Table 5.

The highest overall acceptability was obtained for the frozen AJ samples fermented for 48 h with LUHS122, and a moderate positive correlation was found between the overall acceptability of the AJ and the emotion ‘happy’ (r = 0.7617). A moderate positive correlation was also established between the overall acceptability of the AJ and the emotion ‘surprised’ (r = 0.4888). In contrast to the findings for positive emotions, negative correlations were found between the overall acceptability of the AJ and the negative emotions ‘sad’, ‘angry’, ‘scared’, ‘disgusted’, and ‘contempt’ (r = −0.4325, r = −0.2411, r = −0.2642, r = −0.4212, and r = −0.1282, respectively). The frozen AJ fermented for 48 h with LUHS122 had a pH of 3.96, 14.7% DM, and L*, a*, b* colour characteristics of 50.7, 8.17, and 33.1, respectively (Table 5). However, no correlations were found between the overall acceptability of the AJ and the above-mentioned parameters except for yellowness (b*; r = −0.6791).

The viable LAB count in fermented AJ is shown in Table 6. Frozen AJ fermented for 48 h with LUHS122 showed 7.59 log_10_ CFU mL^−1^ viable LAB count. In comparison above mentioned samples with other samples, similar LAB count in fresh AJ fermented for 48 h with LUHS122 was established (7.44 log_10_ CFU mL^−1^), however, taking into consideration the highest overall acceptability of the frozen samples fermented for 48 h with LUHS122, they were selected for the further experiment.

The saccharide and ethanol concentrations in fermented AJ are shown in Table 7. After 48 h of fermentation, the sugar concentration in most of the AJ samples was reduced (on average, fructose by 2.99%, glucose by 3.26%, and saccharose by 9.92%); the exceptions were fructose and glucose in fresh AJ samples fermented with LUHS210. Moderate positive correlations were established between fructose and glucose concentrations in AJ and overall acceptability (r = 0.3602 and r = 0.3926, respectively), and a moderate negative correlation was found between the overall acceptability of the AJ and ethanol concentration (r = −0.4810).

Comparing non-fermented and fermented AJ, a broader spectrum of pathogen inhibition was shown by fermented AJ samples (Table 8), and the sample with the highest overall acceptability (AJ fermented for 48 h with LUHS122) inhibited 4 out of 10 tested pathogens. Also, reducing the pH and increasing the ethanol concentration of AJ samples increased the spectrum of pathogen inhibition, and moderate negative correlations were found between pH and ethanol concentration and the number of pathogens inhibited (r = −0.4067 and r = −0.5051, respectively).

Finally, frozen AJ fermented for 48 h with LUHS122 was selected for further development of NCC because it showed the highest overall acceptability and induced the greatest intensity of the emotion ‘happy’ as well as containing the highest number of viable LAB, and showing inhibitions properties against *Staphylococcus aureus*, *S. haemolyticus*, *Bacillus subtilis*, and *Streptococcus mutans* strains.

### 3.3. Characteristics of Fermented Apple Juice, Cider, and Vinegar Combinations

During the second stage of the experiment, combinations of AJ, C, and vinegar (V) were developed for the preparation of NCC. The overall acceptability and emotions induced for consumers by the prepared combinations are shown in Table 9. The highest overall acceptability was obtained for the combination prepared from 100 mL of frozen AJ fermented for 48 h with LUHS122 + 100 mL of C-No.3 + 2 mL of V. A very strong positive correlation was established between this combination’s overall acceptability and the emotion ‘happy’ (r = 0.9400). Also, most of the negative emotions (‘sad’, ‘scared’, ‘disgusted’, and ‘contempt’) were negatively correlated with the overall acceptability of the samples (r = −0.5220, r = −0.1730, r = −0.5070, and r = −0.5770, respectively).

Reducing pH and increasing the ethanol concentration tended to decrease the overall acceptability of the combinations (r = −0.3140 and r = −0.7450, respectively) (Table 10). In contrast, increasing DM and the concentration of sugars (fructose, glucose, and saccharose) increased their overall acceptability (r = 0.7080, r = 0.8030, r = 0.7460, and r = 0.7400). Analysis of overall acceptability showed that increasing samples’ a* and b* colour characteristics tended to increase their overall acceptability (r = 0.4020 and r = 0.1940, respectively).

The antimicrobial properties of AJ, C, and V combinations are shown in Table 11. The tested combination inhibited from 3 to 8 of the pathogenic strains tested and the combination with the highest overall acceptability (prepared from 100 mL of frozen AJ fermented for 48 h with LUHS122 + 100 mL of C-No.3 + 2 mL of V) inhibited 8 out of 10 of the pathogens: *Klebsiella pneumoniae*, *Cronobacter sakazakii*, *Acinetobacter baumannii*, *Pseudomonas aeruginosa*, *Staphylococcus aureus*, *S. haemolyticus*, *Bacillus subtilis*, and *Streptococcus mutans*, with inhibition zones of 11.3, 14.2, 11.1, 13.3, 12.2, 14.3, 13.1, and 14.0 mm diameter, respectively.

Finally, for preparation of NCC, the combination consisting of 100 mL of frozen AJ fermented for 48 h with LUHS122 + 100 mL of C-No.3 + 2 mL of V was selected because it showed the highest overall acceptability, induced a high intensity of the emotion ‘happy’ for judges, and showed broad spectrum of pathogenic strain inhibition.

### 3.4. Volatile Compound Profile of Non-Fermented and Fermented Apple Juice and Cider Samples

**Figure 2:**
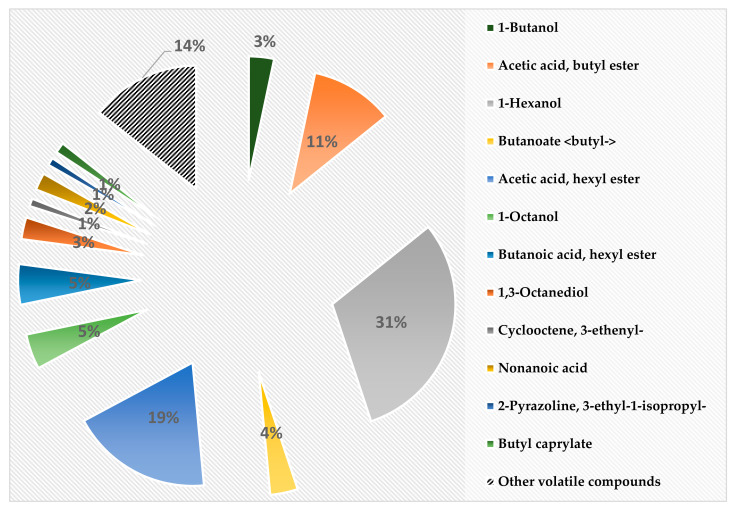
Volatile compound profile (% from the total volatile compounds) of non-fermented frozen apple juice.

The main VC in non-fermented frozen AJ are shown in Figure 2; the whole VC profile is given in Table S1. More than 60% of all the VC in non-fermented frozen AJ were accounted for by acetic acid, butyl ester (10.9%), 1-hexanol (30.7%), and acetic acid, hexyl ester (18.7%). Acetic acid, butyl ester possesses a sharp, etherial, diffusive, fruity banana odour. The odour of 1-hexanol is pungent, etherial, fusel oil, fruity and alcoholic, sweet with a green top note. The odour of acetic acid, hexyl ester is described as green, fruity, sweet, fatty, fresh, apple, and pear. The content of butanoic acid, hexyl ester in non-fermented frozen AJ was higher than 5.0%. The odour of this VC is described as green, fruity, estery, and vegetative with a waxy nuance. Other VC which are shown in Figure 2 were found in quantities lower than 5% but higher than 1% (1-butanol (3.3%), butanoate <butyl-> (3.6%), 1-octanol (4.6%), 1,3-octanediol (2.9%), cyclooctene, 3-ethenyl- (1.0%), nonanoic acid (2.3%), 2-pyrazoline, 3-ethyl-1-isopropyl- (1.0%), and butyl caprylate (1.4%)). The odour of 1-butanol is fusel oil, sweet balsam, and whiskey; butanoate <butyl-> has a pleasant aroma, being used in the flavour industry to create sweet, fruity flavours that are similar to that of pineapple. The odour of 1-octanol is waxy, green, citrus, aldehydic, and floral with a sweet, fatty, coconut nuance. The odour of 1,3-octanediol is musty; that of nonanoic acid is waxy, dirty, and cheesy with a cultured dairy nuance. The odour of butyl caprylate is described as buttery, etherial, herbal, and dank.

The content of the other VC (in total, 14.4% of the whole VC profile), the individual content of which was lower than 1%, are given in Table S1.

The main VC of C samples are shown in Table 12; the whole VC profile is given in Table S2. An increase in the content of 1-butanol, 3-methyl-; 1-hexanol; hexanoic acid; hexanoic acid, ethyl ester; octanoic acid; octanoate <ethyl->; nonanoic acid; 1-decanol; decanoate <ethyl->; and 7,9-di-*tert*-butyl-1-oxaspiro(4,5)deca-6,9-diene-2,8-dione increased the overall acceptability of the C samples and positive correlations were found between overall acceptability and the above-mentioned VC (r = 0.5170, r = 0.8552, r = 0.6128, r = 0.5382, r = 0.6878, r = 0.6066, r = 0.1811, r = 0.1741, r = 0.2913, and r = 0.4030, respectively). 1-Butanol, 3-methyl- possesses a fusel, alcoholic, pungent, etherial, cognac, fruity, banana, and molasses odour. The odour of 1-hexanol is pungent, etherial, fusel oil, fruity, and alcoholic, sweet with a green top note. Hexanoic acid has a sour, fatty, sweaty cheese odour; a description of the odour of hexanoic acid, ethyl ester is given above. Octanoic acid has a fatty, waxy, rancid, oily, vegetable, cheesy odour and octanoate <ethyl-> possesses a waxy, sweet, musty, pineapple, and fruity odour with a creamy, dairy nuance. The odour of nonanoic acid is waxy, dirty, and cheesy with a cultured dairy nuance; that of 1-decanol is fatty, waxy, floral, orange, sweet, clean, and watery. The odour of decanoate <ethyl-> is sweet, waxy, fruity, and apple; and 7,9-di-*tert*-butyl-1-oxaspiro[4,5]deca-6,9-diene-2,8-dione is one of the aroma components in mushrooms.

The main VC in fermented AJ are shown in Table 13; the whole VC profile is given in Table S3. It was established that increasing the content of some of the VC increased the overall acceptability of the samples and positive correlations were found between overall acceptability and 1-butanol (r = 0.3239), butyrate <ethyl-> (r = 0.3239), acetic acid, butyl ester (r = 0.4962), butanoate <butyl-> (r = 0.4668), acetic acid, hexyl ester (r = 0.3018), 1-octanol (r = 0.5051), nonanoic acid (r = 0.2627), 2-pyrazoline, 3-ethyl-1-isopropyl- (r = 0.2488), *n*-decanoic acid (r = 0.8445), and 7,9-di-*tert*-butyl-1-oxaspiro(4,5)deca-6,9-diene-2,8-dione (r = 0.6617). A description of the odours of the VC 1-butanol; acetic acid, butyl ester; butanoate <butyl->; acetic acid, hexyl ester; 1-octanol; and nonanoic acid is given above. In addition. butyrate <ethyl has a sweet, fruity, tutti frutti, lifting, and diffusive odour. 2-Pyrazoline, 3-ethyl-1-isopropyl- has a sharp musty odour like rubbing alcohol and that of n-decanoic acid is described as unpleasant, rancid, sour, fatty, and citrus.

### 3.5. Characteristics of Nutraceutical Chewing Candies Based on Combinations of Fermented Apple Juice, Cider, and Vinegar

The characteristics (overall acceptability, emotions induced for consumers, colour coordinates, and texture) and images of the developed NCC are given in Table 14.

The overall acceptability of the prepared NCC was, on average, 9.03 points, and the highest overall acceptability was obtained for NCC-4 prepared using gelatin for texture formation and stevia as a sweetener. A very strong positive correlation was found between NCC overall acceptability and the emotion ‘happy’ (r = 0.8916). Negative correlations were established between overall acceptability and the negative emotions ‘sad’, ‘angry’, and ‘disgusted’ (r = −0.3693, r = −0.8428, and r = −0.7322, respectively). The highest lightness (L*) was shown by NCC-1 prepared using gelatin and xylitol (50.7 NBS). A very weak positive correlation was found between L* and overall acceptability (r = 0.8916). The highest redness (a*) was shown by NCC-1 (prepared with gelatin and xylitol) and NCC-2 samples (prepared with pectin, xylitol, and lemon juice), and the highest greenness (−a*) by NCC-5 and NCC-6 samples; a moderate negative correlation was found between NCC overall acceptability and a* (r = −0.4563). Also, samples NCC-5 and NCC-6 showed the lowest yellowness (b*) which was, on average, 28.3% lower than that of NCC-1 and NCC-2 formulations and 17.2% lower than that of NCC-3 and NCC-4 formulations. A weak negative correlation was found between NCC overall acceptability and b* (r = −0.1477). The hardest texture was shown by NCC-3, NCC-5, and NCC-6 formulations (on average, 4.03 mJ). Also, different hardness values were obtained for the NCC prepared with gelatin. NCC-1 samples were 20.0% less hard than NCC-4. A weak positive correlation was established between NCC overall acceptability and hardness (r = 0.2266).

## 4. Discussion

Nowadays, healthy lifestyle it is expected to propel the demand for functional food and development of the nutraceuticals market. It is estimated that the global nutraceuticals market will reach USD 658.11 billion by 2028 [25]. After North America, Japan and China are the next largest nutraceutical consumers [26]. Nowadays, consumers are looking for products which are prepared from local natural sources and possess health benefits beyond the basic nutritional value. Such a type of additional diet could be nutraceuticals which are recommended to promote general well-being and prevent illnesses. Globally, nutraceuticals become very important as they are a part of consumers’ daily diet; from this point of view, they generate additional economic prosperity for the region. Taking into consideration that apples are the most popular fruits, applying different bioconversion schemes to their products (juice) could be a very promising technology for local industry to increase their assortment of products and for consumers to get higher-value products for daily consumption. However, new products must possess not just an additional value (possess desirable antimicrobial properties, high viable LAB count, etc.) but also good sensory properties and high overall acceptability. In addition, consumers are looking for healthy products which are marked as ‘without additional sugar’, ‘without chemical preservatives’, etc.

For this reason, first of all in this study the main ingredients were selected according to their sensory properties and the emotions induced for consumers. Standard methods of evaluating the overall acceptability of a food leads to concrete results; however, implicit measures capture especially the total food experience from pre- to post-consumption, which not only relates to the food itself but also to factors such as the physical and social context [27]. In this study, very strong and strong positive correlations were found between the ingredients’ overall acceptability and the emotion ‘happy’ (for AJ and C, and for lactofermented AJ, r = 0.9173 and r = 0.7617, respectively), and negative correlations were established between most of the negative emotions and the overall acceptability of the ingredients. AJ prepared from frozen apples had a higher concentration of fructose, glucose, and saccharose, and these changes were related to higher overall acceptability of this ingredient in comparison with the AJ prepared from fresh apple. Increasing the sugar concentration in frozen AJ is a desirable change because it can lead to a more effective lactofermentation process. Our previous studies showed that the LAB strains used in this study (LUHS122—*L. plantarum* and LUHS210—*L. casei*) have very good (+++) fructose, glucose, and saccharose metabolism as well as good resistance to low environmental pH values [22]. These characteristics of the LAB strains selected for AJ fermentation led to a high number of viable LAB (7.59 log_10_ CFU mL^−1^) in fermented AJ, and fermented AJ had a broader spectrum of pathogen inhibition (inhibited 6 out of 10 tested pathogens) in comparison with non-fermented AJ. AJ is a suitable substrate for LAB fermentation and the viable LAB count in AJ depends on the LAB strain used [28]. But the metabolic pathways of the LAB can be changed in relation to the technological conditions (substrate-specific nutrients and metabolite concentration, duration of the process, etc.). For this reason, control of the end product is very important. The antimicrobial properties of LAB are mainly due to the metabolism of carbohydrates to organic acids (mainly lactic and acetic acids) and lactofermentation results in the production of VC [29]. The fermentation of AJ using LAB strains results in the formation of VC, and seven new alcohols, six new esters, and several ketones and aldehydes in fermented AJ have been established [28]. Our results are in agreement with those of Wu et al. [28] as we found that lactofermentation of AJ products leads to the formation of a specific profile of VC, some of which are related to greater overall acceptability of the fermented products.

Besides that, LAB are Generally Recognized As Safe (GRAS) in the USA and several LAB species fulfil the criteria of Qualified Presumption of Safety (QPS) in Europe [30]. They can possess desirable antimicrobial properties [22]. Our previous studies showed that selected LAB can be used in combination with compounds of plant and animal origin (from berries, fruits, savory plants, bovine colostrum) to increase the antimicrobial activity of each other [31,32,33,34]. Also, apple cider vinegar polyphenols have cytotoxic effects in human urinary bladder cancer cells [35] and the chemoprotective potential of apple components and AJ has been reported [36]. The study of Yagnik et al. showed the antimicrobial potential of apple cider vinegar against *E. coli*, *Staphylococcus aureus*, and *Candida albicans* strains [37]. Hyson [38] summarized and described the relationship between apple compounds and different chronic diseases as well as their relationship with human health. This study showed that fermentation increases the antimicrobial activity of AJ but the combinations of AJ, C, and V developed are more effective as they showed a broader spectrum of pathogen inhibition. In this study, the combination consisting of 100 mL of AJ fermented for 48 h with LUHS122 + 100 mL of C-No.3 + 2 mL of V was selected for preparation of NCC because it showed the highest overall acceptability, induced a high intensity of the emotion ‘happy’ for judges, and inhibited the pathogenic strains *Klebsiella pneumoniae*, *Cronobacter sakazakii*, *Acinetobacter baumannii*, *Pseudomonas aeruginosa*, *Staphylococcus aureus*, *S. haemolyticus*, *Bacillus subtilis*, and *Streptococcus mutans*. This study showed that food biotechnology using different schemes of substrate bioconversion can contribute to the development of higher-value products; future research directions may include the same substrates but different conversion schemes. Finally, the combination of acetic, alcoholic, and lactofermented AJ products leads to the formation of a specific VC profile, and increases the overall acceptability and antimicrobial activity of the products which could be successfully applied in the food and nutraceutical industries.

## 5. Conclusions

AJ prepared from frozen apples showed 11.1% higher overall acceptability (OA) and this could be associated with the 45.9%, 50.4%, and 33.3% higher fructose, glucose, and saccharose concentration, respectively. All the tested C samples inhibited *Bacillus subtilis* and their OA was, on average, 6.6 points. Fermentation with selected LAB increases the OA of AJ and the highest OA was obtained for AJ fermented for 48 h with *L. plantarum* strain LUHS122. A moderate positive correlation was found between the OA of the AJ and the emotion ‘happy’ (r = 0.7617) This sample also showed high viable LAB count (7.59 log_10_ CFU mL^−1^) and inhibited 4 out of 10 tested pathogens. For preparation of NCC, the combination consisting of 50 mL of AJ fermented for 48 h with LUHS122 + 50 mL of C-No.3 + 2 mL of V was selected because it showed the highest OA, induced a high intensity of the emotion ‘happy’ for judges’, and showed a broad spectrum of pathogenic strain inhibition (inhibited 8 out of 10 tested pathogens). Finally, it can be stated that the combination of acetic, alcoholic, and lactofermented AJ products leads to the formation of a specific VC profile, and increases the OA and antimicrobial activity of the products which could be successfully applied in the food and nutraceutical industries as the OA of the prepared NCC was, on average, 9.03 points.

## Figures and Tables

**Table 1:** Nutraceutical chewing candy formulas.

NCC	Apple Juice Products			Texture-Forming Agents		Sweetening Agents		
	FJ-LUHS122, mL	C-No.3, mL	Vinegar, mL	Gelatin, g	Pectin, g	Stevia, µL	Xylitol, g	Lemon Juice, mL
NCC-1	50	-	2	16	-	-	8	-
NCC-2	50	-	2	-	6	-	4	4
NCC-3	50	50	2	16	-	-	8	-
NCC-4	50	50	2	16	-	8	-	-
NCC-5	50	50	-	-	6		8	4
NCC-6	50	50	-	-	6	8		4

NCC—nutraceutical chewing candies; FJ-LUHS122—frozen apple juice fermented for 48 h with *L. plantarum* strain LUHS122; C-No.3—apple cider sample No.3; “-”—was not added.

**Table 2:** Overall acceptability, emotions induced for consumers, and physicochemical parameters of apple juice and cider samples.

Parameters		Frozen Apple Juice	Fresh Apple Juice	C-No.1	C-No.2	C-No.3	C-No.4
OA		9.0 ± 0.3 b	8.0 ± 0.4 a	6.0 ± 0.8 a	7.0 ± 0.5 a,b	7.5 ± 0.6 b	6.0 ± 0.7 a
		**Emotions induced for consumers (from 0 to 1)**					
Neutral		0.891 ± 0.024	0.905 ± 0.018	0.819 ± 0.015	0.931 ± 0.021	0.884 ± 0.014	0.890 ± 0.023
Happy		0.240 ± 0.014	0.180 ± 0.011	0.075 ± 0.005	0.093 ± 0.006	0.117 ± 0.009	0.064 ± 0.004
Sad		0.018 ± 0.001	0.016 ± 0.001	0.014 ± 0.002	0.037 ± 0.002	0.040 ± 0.003	0.045 ± 0.002
Angry		0.003 ± 0.001	0.009 ± 0.002	0.031 ± 0.002	0.028 ± 0.001	0.002 ± 0.001	0.008 ± 0.001
Surprised		0.001 ± 0.001	0.004 ± 0.001	0.013 ± 0.002	0.002 ± 0.001	0.007 ± 0.001	0.005 ± 0.001
Scared		0.0004 ± 0.0001	0.0003 ± 0.0002	0.0003 ± 0.0001	0.0001 ± 0.0001	0.0001 ± 0.0001	0.0002 ± 0.0001
Disgusted		0.007 ± 0.001	0.023 ± 0.002	0.012 ± 0.002	0.014 ± 0.001	0.021 ± 0.002	0.021 ± 0.001
Contempt		0.017 ± 0.001	0.029 ± 0.003	0.014 ± 0.001	0.032 ± 0.002	0.030 ± 0.003	0.025 ± 0.003
Valence		0.037 ± 0.004	0.032 ± 0.004	0.094 ± 0.005	0.064 ± 0.004	0.049 ± 0.003	0.023 ± 0.002
		**Physicochemical parameters of the samples**					
pH		3.82 ± 0.02	3.75 ± 0.01	3.95 ± 0.01	3.92 ± 0.02	3.85 ± 0.02	3.87 ± 0.01
DM, %		21.6 ± 0.2	18.1 ± 0.1	4.80 ± 0.12	4.20 ± 0.14	4.10 ± 0.11	4.60 ± 0.10
L*	**Colour characteristics, NBS**	50.6 ± 2.1	50.7 ± 1.3	89.9 ± 1.2	90.0 ± 0.9	87.6 ± 0.7	89.5 ± 0.6
a*		11.5 ± 1.0	13.3 ± 1.1	−1.39 ± 0.12	−1.4 ± 0.14	−1.91 ± 0.18	−0.960 ± 0.015
b*		36.4 ± 1.2	36.9 ± 0.9	5.63 ± 0.45	6.13 ± 0.31	12.5 ± 0.11	6.07 ± 0.73

OA—overall acceptability; C—apple cider; DM—dry matter; L* lightness; a* redness or −a* greenness; b* yellowness or −b* blueness; NBS—National Bureau of Standards units. a,b—The mean values within a line with different letters are significantly different (*p* ≤ 0.05).

**Table 3:** Saccharide and ethanol concentrations in non-fermented apple juice and cider samples.

		Apple Juice Samples		Apple Cider Samples			
		Frozen Apple Juice	Fresh Apple Juice	C-No.1	C-No.2	C-No.3	C-No.4
Fructose	**Saccharides, g 100 g^−1^**	15.1 ± 0.11	8.17 ± 0.08	nd	nd	0.781 ± 0.017	0.490 ± 0.014
Glucose		3.87 ± 0.19	1.92 ± 0.16	nd	nd	nd	nd
Saccharose		2.28 ± 0.19	1.52 ± 0.06	nd	nd	nd	nd
Ethanol, g 100 g^−1^		nd	nd	2.94 ± 0.08	2.44 ± 0.10	2.43 ± 0.11	2.68 ± 0.23

C—apple cider; nd—not detectable.

**Table 4:** Antimicrobial properties of apple juice and cider.

Pathogens	Frozen Apple Juice	Fresh Apple Juice	C-No.1	C-No.2	C-No.3	C-No.4
	Diameter of Inhibition Zone, mm					
*Escherichia coli*	nd	nd	nd	nd	nd	nd
*Klebsiella pneumoniae*	nd	nd	nd	nd	nd	nd
*Salmonella enterica*	nd	nd	nd	nd	nd	nd
*Cronobacter sakazakii*	nd	nd	nd	nd	nd	nd
*Acinetobacter baumannii*	nd	nd	nd	nd	nd	nd
*Pseudomonas aeruginosa*	nd	nd	nd	nd	nd	nd
*Staphylococcus aureus*	nd	nd	nd	nd	nd	nd
*S. haemolyticus*	nd	nd	nd	nd	nd	nd
*Bacillus subtilis*	11.2 ± 0.2	8.34 ± 0.29	8.10 ± 0.31	nd	nd	nd
*Streptococcus mutans*	nd	nd	nd	nd	nd	nd

C—apple cider; nd—not detectable.

**Table 5:** Overall acceptability, emotions induced for consumers, and physicochemical properties of fermented apple juice samples.

Parameters		Frozen Apple Juice				Fresh Apple Juice			
		Lactic Acid Bacteria Strain Used for Fermentation							
		LUHS122		LUHS210		LUHS122		LUHS210	
		Duration of Fermentation, h							
		24	48	24	48	24	48	24	48
OA		7.51 ± 0.65 a	10.0 ± 0.97 c	6.52 ± 0.58 a	8.01 ± 0.71 a,b	7.03 ± 0.69 a	8.01 ± 0.74 a,b	6.03 ± 0.62 a	7.04 ± 0.67 a
		**Emotions induced for consumers (from 0 to 1)**							
Neutral		0.841 ± 0.073 b	0.811 ± 0.061 b	0.894 ± 0.054 b	0.859 ± 0.047 b	0.672 ± 0.033 a	0.836 ± 0.041 b	0.911 ± 0.086 b	0.830 ± 0.059 b
Happy		0.005 ± 0.002 b	0.055 ± 0.004 d	0.001 ± 0.001 a	0.001 ± 0.001 a	0.021 ± 0.002 c	0.002 ± 0.001 a	0.001 ± 0.001 a	0.008 ± 0.003 b
Sad		0.078 ± 0.006 f	0.007 ± 0.002 a	0.026 ± 0.003 c	0.018 ± 0.002 b	0.042 ± 0.003 e	0.009 ± 0.002 a	0.032 ± 0.003 d	0.043 ± 0.004 e
Angry		0.002 ± 0.001 a	0.002 ± 0.001 a	0.004 ± 0.002 a	0.001 ± 0.001 a	0.023 ± 0.002 b	0.005 ± 0.003 a	0.004 ± 0.003 a	0.003 ± 0.001 a
Surprised		0.028 ± 0.002 c	0.048 ± 0.003 e	0.034 ± 0.002 d	0.017 ± 0.002 b	0.008 ± 0.001 a	0.020 ± 0.002 b	0.025 ± 0.003 b,c	0.026 ± 0.002 c
Scared		0.004 ± 0.001 a,b	0.001 ± 0.001 a	0.002 ± 0.001 a	0.002 ± 0.001 a	0.001 ± 0.001 a	0.003 ± 0.002 a	0.003 ± 0.002 a	0.001 ± 0.001 a
Disgusted		0.007 ± 0.002 b	0.001 ± 0.001 a	0.016 ± 0.003 c	0.006 ± 0.002 b	0.017 ± 0.002 c	0.004 ± 0.001 b	0.001 ± 0.001 a	0.023 ± 0.002 c,d
Contempt		0.020 ± 0.003 d	0.013 ± 0.001 b	0.003 ± 0.001 a	0.009 ± 0.002 b	0.015 ± 0.001 b,c	0.021 ± 0.002 d	0.020 ± 0.001 d	0.037 ± 0.004 e
Valence		0.074 ± 0.005 c	0.145 ± 0.013 e	0.018 ± 0.002 a	0.091 ± 0.007 d	0.188 ± 0.015 f	0.095 ± 0.011 d	0.035 ± 0.003 b	0.105 ± 0.011 d
		**Physicochemical parameters of the samples**							
pH		4.55 ± 0.02 g	3.96 ± 0.01 d	3.90 ± 0.02 c	3.88 ± 0.02 c	4.07 ± 0.01 f	3.59 ± 0.01 a	4.02 ± 0.02 e	3.82 ± 0.01 b
DM, %		17.8 ± 0.5 f	14.7 ± 0.1 e	11.4 ± 0.1 c	14.9 ± 0.1 e	11.6 ± 0.1 c	6.31 ± 0.05 a	11.9 ± 0.1 c,d	7.74 ± 0.08 b
L*	**Colour characteristics, NBS**	54.4 ± 0.5 c	50.7 ± 0.4 a	51.8 ± 0.2 b	53.7 ± 0.3 c	55.2 ± 0.3 c	58.3 ± 0.4 d	54.0 ± 0.4 c	51.9 ± 0.4 b
a*		6.41 ± 0.12 c	8.17 ± 0.17 e	8.69 ± 0.14 f	6.65 ± 0.16 c	5.59 ± 0.09 b	2.84 ± 0.11 a	7.70 ± 0.17 d	7.48 ± 0.22 d
b*		34.6 ± 0.2 c	33.1 ± 0.1 a	33.9 ± 0.3 b	34.7 ± 0.2 c	36.0 ± 0.1 d	35.1 ± 0.2 c	36.3 ± 0.3 d	34.7 ± 0.2 c

OA—overall acceptability; LUHS122—*L. plantarum* strain LUHS122; LUHS210—*L. casei* strain LUHS210; DM—dry matter; L* lightness; a* redness or −a* greenness; b* yellowness or −b* blueness; NBS—National Bureau of Standards units. a–f—The mean values within a line with different letters are significantly different (*p* ≤ 0.05).

**Table 6:** Viable lactic acid bacteria count in fermented apple juice samples.

Parameters	Frozen Apple Juice				Fresh Apple Juice			
	Lactic Acid Bacteria Strain Used for Fermentation							
	LUHS122		LUHS210		LUHS122		LUHS210	
	Duration of Fermentation, h							
	24	48	24	48	24	48	24	48
LAB count, log_10_ CFU g^−1^	7.25 ± 0.22 c	7.59 ± 0.11 d	7.14 ± 0.14 b	7.11 ± 0.13 b	6.61 ± 0.16 a	7.44 ± 0.13 c,d	7.09 ± 0.09 b	7.08 ± 0.21 b

LAB—lactic acid bacteria; CFU—colony-forming units; LUHS122—*L. plantarum* strain LUHS122; LUHS210—*L. casei* strain LUHS210. a–d—The mean values within a line with different letters are significantly different (*p* ≤ 0.05).

**Table 7:** Saccharide and ethanol concentrations in fermented apple juice.

Parameters	Frozen Apple Juice				Fresh Apple Juice			
	Lactic Acid Bacteria Strain Used for Fermentation							
	LUHS122		LUHS210		LUHS122		LUHS210	
	Duration of Fermentation, h							
	24	48	24	48	24	48	24	48
Fructose, g 100 g^−1^	11.2 ± 0.10 e	10.9 ± 0.09 d	11.3 ± 0.13 e	10.9 ± 0.11 d	6.85 ± 0.06 b	6.66 ± 0.05 a	7.23 ± 0.07 c	7.21 ± 0.05 c
Glucose, g 100 g^−1^	2.88 ± 0.15 b	2.80 ± 0.18 b	2.86 ± 0.16 b	2.76 ± 0.13 b	1.60 ± 0.11 a	1.55 ± 0.10 a	1.64 ± 0.14 a	1.58 ± 0.13 a
Saccharose, g 100 g^−1^	1.47 ± 0.12 d	1.34 ± 0.10 d	1.43 ± 0.12 d	1.30 ± 0.11 d	0.980 ± 0.018 b	0.880 ± 0.017 a	1.04 ± 0.10 c	0.920 ± 0.021 a
Ethanol, g 100 g^−1^	0.008 ± 0.002 b	0.002 ± 0.001 a	0.001 ± 0.001 a	0.003 ± 0.001 a	0.002 ± 0.001 a	0.001 ± 0.001 a	0.013 ± 0.002 c	0.006 ± 0.002 b

LUHS122—*L. plantarum* strain LUHS122; LUHS210—*L. casei* strain LUHS210. a–e—The mean values within a line with different letters are significantly different (*p* ≤ 0.05).

**Table 8:** Antimicrobial activity of fermented apple juice samples against opportunistic pathogenic microorganisms.

Pathogens	Frozen Apple Juice				Fresh Apple Juice			
	Lactic Acid Bacteria Strain Used for Fermentation							
	LUHS122		LUHS210		LUHS122		LUHS210	
	Duration of Fermentation, h							
	24	48	24	48	24	48	24	48
	Diameter of Inhibition Zone, mm							
*Escherichia coli*	nd	nd	nd	nd	nd	nd	nd	nd
*Klebsiella pneumoniae*	nd	nd	nd	nd	nd	9.2 ± 0.3	nd	nd
*Salmonella enterica*	nd	nd	nd	nd	nd	nd	nd	nd
*Cronobacter sakazakii*	11.1 ± 0.4	nd	nd	nd	10.3 ± 0.3	11.5 ± 0.2	nd	nd
*Acinetobacter baumannii*	nd	nd	nd	nd	nd	nd	nd	nd
*Pseudomonas aeruginosa*	nd	nd	nd	nd	8.2 ± 0.1	nd	nd	nd
*Staphylococcus aureus*	nd	9.1 ± 0.1	nd	nd	nd	12.3 ± 0.2	nd	nd
*S. haemolyticus*	nd	9.2 ± 0.2	nd	nd	nd	12.1 ± 0.3	nd	10.2 ± 0.4
*Bacillus subtilis*	9.2 ± 0.2	12.1 ± 0.3	nd	10.5 ± 0.1	12.1 ± 0.2	13.2 ± 0.2	nd	10.4 ± 0.5
*Streptococcus mutans*	nd	14.5 ± 0.4	nd	12.3 ± 0.2	nd	22.1 ± 0.3	nd	16.2 ± 0.3

LUHS122—*L. plantarum* strain LUHS122; LUHS210—*L. casei* strain LUHS210; nd—not detectable.

**Table 9:** Overall acceptability and emotions induced for consumers by apple juice, cider, and vinegar combinations.

Apple Juice, Cider, and Vinegar Combinations	OA	Emotions Induced for Consumers (from 0 to 1)								
		Neutral	Happy	Sad	Angry	Surprised	Scared	Disgusted	Contempt	Valence
24 h-C-No.1(100/100)-FJLUHS122-V	7.83 ± 0.71	0.761 ± 0.051	0.080 ± 0.007	0.026 ± 0.003	0.014 ± 0.001	0.011 ± 0.001	0.001 ± 0.001	0.006 ± 0.002	0.001 ± 0.001	0.038 ± 0.002
48 h-C-No.1(100/100)-FJLUHS122-V	8.00 ± 0.79	0.872 ± 0.014	0.103 ± 0.010	0.013 ± 0.003	0.034 ± 0.002	0.035 ± 0.001	0.019 ± 0.001	0.003 ± 0.001	0.002 ± 0.001	0.044 ± 0.003
24 h-C-No.1(50/150)-FJLUHS122-V	5.83 ± 0.48	0.854 ± 0.021	0.045 ± 0.005	0.012 ± 0.002	0.027 ± 0.001	0.040 ± 0.005	0.004 ± 0.002	0.004 ± 0.001	0.002 ± 0.001	0.025 ± 0.002
48 h-C-No.1(50/150)-FJLUHS122-V	6.16 ± 0.59	0.747 ± 0.012	0.074 ± 0.018	0.015 ± 0.003	0.041 ± 0.002	0.021 ± 0.001	0.009 ± 0.001	0.008 ± 0.001	0.014 ± 0.001	0.039 ± 0.003
24 h-C-No.2(100/100)-FJLUHS122-V	8.00 ± 0.73	0.832 ± 0.026	0.109 ± 0.010	0.037 ± 0.004	0.078 ± 0.005	0.004 ± 0.002	0.016 ± 0.002	0.006 ± 0.001	0.001 ± 0.001	0.086 ± 0.004
48 h-C-No.2(100/100)-FJLUHS122-V	7.51 ± 0.64	0.871 ± 0.016	0.093 ± 0.008	0.012 ± 0.001	0.042 ± 0.003	0.013 ± 0.001	0.020 ± 0.002	0.005 ± 0.001	0.003 ± 0.001	0.046 ± 0.003
24 h-C-No.2(50/150)-FJLUHS122-V	6.31 ± 0.58	0.898 ± 0.016	0.068 ± 0.011	0.003 ± 0.001	0.069 ± 0.007	0.002 ± 0.001	0.009 ± 0.001	0.001 ± 0.001	0.002 ± 0.001	0.044 ± 0.003
48 h-C-No.2(50/150)-FJLUHS122-V	6.33 ± 0.57	0.908 ± 0.029	0.052 ± 0.006	0.015 ± 0.001	0.022 ± 0.001	0.019 ± 0.002	0.011 ± 0.003	0.001 ± 0.001	0.005 ± 0.002	0.040 ± 0.003
24 h-C-No.3(100/100)-FJLUHS122-V	8.17 ± 0.73	0.914 ± 0.015	0.114 ± 0.011	0.002 ± 0.001	0.065 ± 0.005	0.009 ± 0.004	0.016 ± 0.001	0.001 ± 0.001	0.005 ± 0.001	0.059 ± 0.004
48 h-C-No.3(100/100)-FJLUHS122-V	9.01 ± 0.85	0.910 ± 0.017	0.153 ± 0.013	0.002 ± 0.001	0.039 ± 0.004	0.015 ± 0.002	0.007 ± 0.001	0.001 ± 0.001	0.004 ± 0.001	0.070 ± 0.006
24 h-C-No.3(50/150)-FJLUHS122-V	7.01 ± 0.59	0.907 ± 0.019	0.094 ± 0.008	0.006 ± 0.002	0.054 ± 0.004	0.006 ± 0.002	0.001 ± 0.001	0.005 ± 0.002	0.004 ± 0.002	0.040 ± 0.005
48 h-C-No.3(50/150)-FJLUHS122-V	7.10 ± 0.62	0.849 ± 0.028	0.089 ± 0.009	0.010 ± 0.001	0.010 ± 0.002	0.031 ± 0.003	0.010 ± 0.001	0.003 ± 0.001	0.003 ± 0.001	0.028 ± 0.003
24 h-C-No.4(100/100)-FJLUHS122-V	8.16 ± 0.63	0.860 ± 0.036	0.121 ± 0.021	0.021 ± 0.002	0.027 ± 0.003	0.025 ± 0.004	0.001 ± 0.001	0.045 ± 0.003	0.001 ± 0.001	0.006 ± 0.002
48 h-C-No.4(100/100)-FJLUHS122-V	8.50 ± 0.71	0.901 ± 0.046	0.130 ± 0.010	0.002 ± 0.001	0.053 ± 0.004	0.013 ± 0.004	0.001 ± 0.001	0.001 ± 0.001	0.005 ± 0.002	0.033 ± 0.004
24 h-C-No.4(50/150)-FJLUHS122-V	7.16 ± 0.59	0.830 ± 0.041	0.093 ± 0.010	0.006 ± 0.002	0.005 ± 0.001	0.010 ± 0.001	0.008 ± 0.002	0.018 ± 0.001	0.007 ± 0.001	0.013 ± 0.002
48 h-C-No.4(50/150)-FJLUHS122-V	5.16 ± 0.43	0.695 ± 0.036	0.042 ± 0.003	0.695 ± 0.049	0.001 ± 0.001	0.013 ± 0.002	0.020 ± 0.001	0.394 ± 0.021	0.045 ± 0.003	0.212 ± 0.018

OA—overall acceptability; 24 h and 48 h—duration of fermentation; C—apple cider; 100/100 and 50/150—ratio of cider/fermented apple juice; FJ—frozen apple juice; LUHS122—fermented with *L. plantarum* strain LUHS122; V—apple vinegar culture (2 mL).

**Table 10:** Physicochemical parameters of apple juice, cider, and vinegar combinations.

Apple Juice, Cider, and Vinegar Combinations	pH	DM, %	NBS Colour Coordinates			Ethanol, g 100 g^−1^	Sugar Concentration, g 100 g^−1^		
			L*	a*	b*		F	G	S
24 h-C-No.1(100/100)-FJLUHS122-V	3.81 ± 0.01	9.80 ± 0.10	60.9 ± 0.2	1.44 ± 0.02	35.3 ± 0.2	2.48 ± 0.19	7.81 ± 0.53	2.44 ± 0.18	1.26 ± 0.11
48 h-C-No.1(100/100)-FJLUHS122-V	3.90 ± 0.02	13.5 ± 0.1	36.3 ± 0.1	1.56 ± 0.04	34.6 ± 0.1	2.50 ± 0.14	7.40 ± 0.32	2.46 ± 0.19	1.04 ± 0.10
24 h-C-No.1(50/150)-FJLUHS122-V	3.83 ± 0.01	9.01 ± 0.09	61.1 ± 0.2	0.841 ± 0.013	37.2 ± 0.2	2.68 ± 0.21	3.91 ± 0.29	1.56 ± 0.14	0.950 ± 0.022
48 h-C-No.1(50/150)-FJLUHS122-V	3.92 ± 0.01	9.10 ± 0.08	64.3 ± 0.2	−3.00 ± 0.23	29.9 ± 0.1	3.11 ± 0.26	3.92 ± 0.27	1.57 ± 0.13	0.800 ± 0.041
24 h-C-No.2(100/100)-FJLUHS122-V	3.49 ± 0.02	13.4 ± 0.1	67.1 ± 0.2	−1.56 ± 0.15	27.5 ± 0.1	2.21 ± 0.18	7.46 ± 0.36	2.31 ± 0.21	1.21 ± 0.10
48 h-C-No.2(100/100)-FJLUHS122-V	3.82 ± 0.01	13.7 ± 0.1	55.3 ± 0.4	3.16 ± 0.11	34.9 ± 0.1	2.22 ± 0.15	7.62 ± 0.53	2.49 ± 0.22	1.09 ± 0.13
24 h-C-No.2(50/150)-FJLUHS122-V	3.85 ± 0.01	8.20 ± 0.07	69.2 ± 0.1	−2.37 ± 0.21	25.9 ± 0.2	2.78 ± 0.18	3.15 ± 0.13	1.31 ± 0.10	0.860 ± 0.023
48 h-C-No.2(50/150)-FJLUHS122-V	3.86 ± 0.02	9.20 ± 0.08	63.3 ± 0.1	−1.72 ± 0.14	32.6 ± 0.2	2.72 ± 0.20	3.40 ± 0.26	1.48 ± 0.13	0.810 ± 0.052
24 h-C-No.3(100/100)-FJLUHS122-V	3.80 ± 0.02	10.6 ± 0.1	38.9 ± 0.2	3.30 ± 0.13	35.5 ± 0.2	1.66 ± 0.15	7.21 ± 0.31	2.18 ± 0.19	1.27 ± 0.14
48 h-C-No.3(100/100)-FJLUHS122-V	3.83 ± 0.02	13.7 ± 0.1	59.9 ± 0.2	0.070 ± 0.06	31.7 ± 0.1	1.09 ± 0.03	7.53 ± 0.41	2.34 ± 0.16	1.12 ± 0.11
24 h-C-No.3(50/150)-FJLUHS122-V	3.87 ± 0.01	8.3 ± 0.07	63.9 ± 0.1	0.300 ± 0.014	32.2 ± 0.2	2.57 ± 0.12	3.73 ± 0.22	1.36 ± 0.14	0.960 ± 0.051
48 h-C-No.3(50/150)-FJLUHS122-V	3.86 ± 0.01	9.00 ± 0.08	61.3 ± 0.2	−0.430 ± 0.012	31.4 ± 0.1	2.31 ± 0.15	3.12 ± 0.21	1.23 ± 0.14	0.750 ± 0.028
24 h-C-No.4(100/100)-FJLUHS122-V	3.80 ± 0.01	10.5 ± 0.1	59.4 ± 0.1	3.07 ± 0.05	35.3 ± 0.2	1.89 ± 0.09	7.55 ± 0.26	2.30 ± 0.19	1.21 ± 0.10
48 h-C-No.4(100/100)-FJLUHS122-V	3.84 ± 0.02	13.7 ± 0.1	56.9 ± 0.2	2.47 ± 0.04	34.2 ± 0.2	2.03 ± 0.16	7.99 ± 0.38	2.48 ± 0.21	1.08 ± 0.11
24 h-C-No.4(50/150)-FJLUHS122-V	3.88 ± 0.02	9.1 ± 0.24	64.3 ± 0.3	0.250 ± 0.06	31.9 ± 0.2	2.44 ± 0.21	3.74 ± 0.29	1.44 ± 0.11	0.880 ± 0.063
48 h-C-No.4(50/150)-FJLUHS122-V	3.86 ± 0.02	9.50 ± 0.08	38.2 ± 0.2	1.68 ± 0.11	32.1 ± 0.1	2.39 ± 0.12	4.32 ± 0.25	1.63 ± 0.14	0.850 ± 0.042

DM—dry matter; L* lightness; a* redness or −a* greenness; b* yellowness or −b* blueness; NBS—National Bureau of Standards units; F—fructose; G—glucose; S—saccharose; 24 h and 48 h—duration of fermentation; C—apple cider; 100/100 and 50/150—ratio of cider/fermented apple juice; FJ—frozen apple juice; LUHS122—fermented with *L. plantarum* strain LUHS122; V—apple vinegar culture (2 mL).

**Table 11:** Antimicrobial properties of apple juice, cider, and vinegar combinations.

Apple Juice, Cider, and Vinegar Combinations	Pathogens									
	*Escherichia coli*	*Klebsiella pneumoniae*	*Salmonella enterica*	*Cronobacter sakazakii*	*Acinetobacter baumannii*	*Pseudomonas aeruginosa*	*Staphylococcus aureus*	*S. haemolyticus*	*Bacillus subtilis*	*Streptococcus mutans*
	Diameter of Inhibition Zone, mm									
24 h-C-No.1(100/100)-FJLUHS122-V	12.1 ± 0.1	10.2 ± 0.2	nd	nd	11.1 ± 0.3	10.0 ± 0.3	nd	11.0 ± 0.2	11.3 ± 0.2	nd
48 h-C-No.1(100/100)-FJLUHS122-V	13.3 ± 0.3	12.4 ± 0.2	nd	14.2 ± 0.4	10.0 ± 0.2	11.1 ± 0.3	14.3 ± 0.4	11.2 ± 0.4	11.1 ± 0.3	nd
24 h-C-No.1(50/150)-FJLUHS122-V	13.2 ± 0.3	11.0 ± 0.2	14.1 ± 0.3	12.3 ± 0.4	12.1 ± 0.1	13.0 ± 0.3	nd	nd	14.2 ± 0.2	nd
48 h-C-No.1(50/150)-FJLUHS122-V	14.1 ± 0.2	11.1 ± 0.1	nd	14.3 ± 0.2	nd	10.2 ± 0.1	14.0 ± 0.2	9.0 ± 0.3	11.1 ± 0.1	nd
24 h-C-No.2(100/100)-FJLUHS122-V	13.3 ± 0.2	nd	nd	nd	10.0 ± 0.1	nd	nd	nd	10.2 ± 0.2	nd
48 h-C-No.2(100/100)-FJLUHS122-V	12.3 ± 0.1	10.0 ± 0.2	nd	10.1 ± 0.3	nd	nd	nd	nd	13.3 ± 0.4	nd
24 h-C-No.2(50/150)-FJLUHS122-V	13.2 ± 0.3	10.3 ± 0.2	nd	12.4 ± 0.3	10.5 ± 0.4	nd	nd	nd	11.4 ± 0.3	nd
48 h-C-No.2(50/150)-FJLUHS122-V	10.1 ± 0.2	10.2 ± 0.1	nd	10.4 ± 0.3	nd	nd	nd	nd	10.2 ± 0.2	nd
24 h-C-No.3(100/100)-FJLUHS122-V	nd	11.1 ± 0.2	12.2 ± 0.3	13.1 ± 0.2	12.0 ± 0.2	12.0 ± 0.3	9.3 ± 0.1	12.1 ± 0.2	10.1 ± 0.3	nd
48 h-C-No.3(100/100)-FJLUHS122-V	nd	11.3 ± 0.2	nd	14.2 ± 0.3	11.1 ± 0.1	13.3 ± 0.2	12.2 ± 0.3	14.3 ± 0.1	13.1 ± 0.2	14.0 ± 0.2
24 h-C-No.3(50/150)-FJLUHS122-V	nd	12.3 ± 0.3	13.2 ± 0.1	13.0 ± 0.2	13.1 ± 0.3	13.0 ± 0.1	nd	12.1 ± 0.2	9.2 ± 0.1	nd
48 h-C-No.3(50/150)-FJLUHS122-V	nd	10.2 ± 0.1	nd	14.1 ± 0.2	11.1 ± 0.3	11.0 ± 0.2	11.3 ± 0.3	nd	10.2 ± 0.2	13.2 ± 0.2
24 h-C-No.4(100/100)-FJLUHS122-V	nd	9.3 ± 0.2	13.2 ± 0.1	11.2 ± 0.3	9.0 ± 0.3	13.2 ± 0.2	nd	10.1 ± 0.1	11.3 ± 0.3	nd
48 h-C-No.4(100/100)-FJLUHS122-V	nd	10.3 ± 0.2	nd	14.2 ± 0.1	9.3 ± 0.1	10.2 ± 0.2	10.3 ± 0.3	12.5 ± 0.2	10.3 ± 0.1	12.1 ± 0.2
24 h-C-No.4(50/150)-FJLUHS122-V	nd	10.2 ± 0.2	11.0 ± 0.1	10.1 ± 0.2	12.2 ± 0.2	10.1 ± 0.1	nd	12.0 ± 0.3	12.3 ± 0.2	nd
48 h-C-No.4(50/150)-FJLUHS122-V	nd	12.3 ± 0.2	nd	12.2 ± 0.3	12.3 ± 0.2	10.1 ± 0.1	nd	14.2 ± 0.2	12.3 ± 0.3	14.1 ± 0.1

24 h and 48 h—duration of fermentation; C—apple cider; 100/100 and 50/150—ratio of cider/fermented apple juice; FJ—frozen apple juice; LUHS122—fermented with *L. plantarum* strain LUHS122; V—apple vinegar culture (2 mL); nd—not detectable.

**Table 12:** Volatile compound profile (% from the total volatile compounds) of cider samples.

RT, min	Volatile Compound, % from the Total Volatile Compounds	C-No.1	C-No.2	C-No.3	C-No.4
4.373	3-methylbutan-1-ol	27.9 ± 0.3	22.9 ± 0.2	29.1 ± 0.2	18.9 ± 0.2
6.159	Ethyl 2-hydroxypropanoate	nd	2.06 ± 0.18	nd	1.32 ± 0.24
7.594	1-Hexanol	6.41 ± 0.27	5.97 ± 0.31	9.22 ± 0.34	5.27 ± 0.30
10.492	Hexanoic acid	1.45 ± 0.14	1.58 ± 0.13	1.66 ± 0.11	nd
11.022	Hexanoic acid, ethyl ester	1.99 ± 0.17	4.25 ± 0.29	3.86 ± 0.22	3.78 ± 0.29
12.803	1-Octanol	2.13 ± 0.16	1.27 ± 0.17	nd	1.09 ± 0.09
13.911	Phenethyl alcohol	11.6 ± 0.2	7.93 ± 0.42	9.99 ± 0.38	8.47 ± 0.38
15.383	Butanedioic acid, diethyl ester	8.76 ± 0.09	4.78 ± 0.31	nd	4.78 ± 0.20
15.472	Octanoic acid	nd	7.11 ± 0.36	4.04 ± 0.23	nd
15.69	Butanoic acid, hexyl ester	0.101 ± 0.009	nd	nd	12.5 ± 0.08
15.783	Ethyl octanoate	1.78 ± 0.15	8.04 ± 0.43	6.77 ± 0.41	5.89 ± 0.29
16.158	4-Octanol, 2,4-dimethyl-	1.40 ± 0.12	nd	nd	nd
17.174	1,3-Octanediol	3.46 ± 0.21	0.780 ± 0.027	1.47 ± 0.26	0.541 ± 0.031
17.338	Nonanoic acid	4.77 ± 0.32	2.07 ± 0.14	6.47 ± 0.32	5.40 ± 0.32
17.444	1-Decanol	nd	2.13 ± 0.20	nd	nd
19.322	3-Allyl-6-methoxyphenol	3.30 ± 0.29	0.630 ± 0.011	nd	0.376 ± 0.025
19.358	*n*-Decanoic acid	1.96 ± 0.15	6.91 ± 0.30	7.45 ± 0.51	11.3 ± 0.1
19.974	Ethyl decanoate	0.622 ± 0.041	6.16 ± 0.41	2.86 ± 0.19	3.65 ± 0.26
21.576	2,6-*bis*(1,1-dimethylethyl)-2,5-cyclohexadiene-1,4-dione	2.29 ± 0.13	1.43 ± 0.11	1.52 ± 0.10	1.45 ± 0.11
22.275	2,4-*bis*(1,1-dimethylethyl)phenol	5.65 ± 0.28	3.52 ± 0.15	2.50 ± 0.21	3.78 ± 0.25
29.199	7,9-Di-*tert*-butyl-1-oxaspiro(4,5)deca-6,9-diene-2,8-dione	3.41 ± 0.25	2.34 ± 0.21	3.75 ± 0.26	2.58 ± 0.19

RT—retention time; C—cider samples; nd—not detectable.

**Table 13:** Volatile compound profile (% from the total volatile compounds) of fermented apple juice.

RT, min	Volatile Compound, % from the Total Volatile Compounds	Frozen Apple Juice				Fresh Apple Juice			
		Lactic Acid Bacteria Strain Used for Fermentation							
		LUHS122		LUHS210		LUHS122		LUHS210	
		Duration of Fermentation, h							
		24	48	24	48	24	48	24	48
2.471	Ethyl acetate	nd	nd	nd	nd	nd	2.83 ± 0.26	2.38 ± 0.21	nd
3.05	1-Butanol	3.58 ± 0.21	**4.50** ± 0.29	5.59 ± 0.39	4.99 ± 0.36	1.30 ± 0.11	1.47 ± 0.12	1.57 ± 0.14	1.90 ± 0.17
4.373	3-methylbutan-1-ol	0.884 ± 0.041	**0.620** ± 0.035	2.36 ± 0.21	1.87 ± 0.14	1.73 ± 0.16	4.07 ± 0.25	8.33 ± 0.25	10.3 ± 0.1
5.861	Ethyl butyrate	1.04 ± 0.03	**0.811** ± 0.41	nd	nd	0.816 ± 0.045	1.06 ± 0.09	nd	nd
6.21	Acetic acid, butyl ester	11.6 ± 0.2	**10.6** ± 0.1	1.80 ± 0.16	1.78 ± 0.16	9.17 ± 0.08	1.97 ± 0.14	0.991 ± 0.028	1.10 ± 0.11
7.594	1-Hexanol	36.4 ± 0.3	**40.5** ± 0.3	49.1 ± 0.3	44.8 ± 0.3	33.1 ± 0.3	38.7 ± 0.3	36.9 ± 0.2	40.2 ± 0.4
10.492	Hexanoic acid	0.913 ± 0.059	**1.07** ± 0.09	1.25 ± 0.11	1.11 ± 0.09	1.05 ± 0.07	1.48 ± 0.11	1.44 ± 0.11	1.67 ± 0.14
10.947	Butyl butanoate	2.48 ± 0.23	**2.20** ± 0.21	0.903 ± 0.060	0.989 ± 0.037	2.52 ± 0.23	1.44 ± 0.12	0.514 ± 0.048	nd
11.022	Hexanoic acid, ethyl ester	nd	nd	nd	nd	nd	nd	1.45 ± 0.13	1.69 ± 0.15
11.402	Acetic acid, hexyl ester	8.41 ± 0.34	**5.54** ± 0.36	3.18 ± 0.15	2.98 ± 0.23	6.38 ± 0.41	3.94 ± 0.32	2.72 ± 0.23	3.07 ± 0.29
11.775	2-ethyl-1-Hexanol	0.663 ± 0.041	**0.599** ± 0.41	0.871 ± 0.036	9.41 ± 0.34	8.15 ± 0.39	8.88 ± 0.51	8.13 ± 0.31	8.87 ± 0.42
12.803	1-Octanol	4.30 ± 0.31	**4.07** ± 0.29	3.98 ± 0.27	4.09 ± 0.36	3.24 ± 0.26	2.67 ± 0.19	1.23 ± 0.10	1.40 ± 0.12
13.65	*cis*-1,2-Cyclohexanedimethanol	0.726 ± 0.028	**0.709** ± 0.035	0.951 ± 0.042	0.902 ± 0.039	0.930 ± 0.057	0.902 ± 0.052	0.881 ± 0.030	1.10 ± 0.09
13.911	Phenethyl alcohol	0.661 ± 0.031	**0.835** ± 0.043	0.963 ± 0.053	0.496 ± 0.028	1.41 ± 0.35	2.62 ± 0.18	3.48 ± 0.31	3.81 ± 0.34
15.783	Ethyl octanoate	0.116 ± 0.011	nd	0.252 ± 0.020	0.233 ± 0.015	0.267 ± 0.016	0.639 ± 0.025	2.26 ± 0.19	2.66 ± 0.23
17.174	1,3-Octanediol	2.63 ± 0.24	**3.13** ± 0.24	5.48 ± 0.29	5.04 ± 0.41	4.33 ± 0.20	3.23 ± 0.21	4.90 ± 0.26	5.80 ± 0.31
17.338	Nonanoic acid	4.48 ± 0.33	**5.53** ± 0.40	5.20 ± 0.31	5.37 ± 0.37	4.49 ± 0.31	6.32 ± 0.36	5.52 ± 0.41	2.19 ± 0.19
17.807	3-ethyl-1-isopropyl-2-pyrazoline	1.45 ± 0.12	**1.30** ± 0.12	1.62 ± 0.11	nd	nd	nd	nd	nd
19.322	3-Allyl-6-methoxyphenol	1.91 ± 0.17	nd	nd	nd	3.50 ± 0.25	2.12 ± 0.19	3.34 ± 0.29	nd
19.358	*n*-Decanoic acid	nd	**1.66** ± 0.14	nd	1.02 ± 0.09	nd	nd	nd	nd
22.275	2,4-*bis*(1,1-dimethylethyl)phenol	3.16 ± 0.26	**2.59** ± 0.23	2.17 ± 0.18	1.84 ± 0.16	3.87 ± 0.28	3.73 ± 0.31	2.31 ± 0.21	2.19 ± 0.18
29.199	7,9-Di-*tert*-butyl-1-oxaspiro (4,5)deca-6,9-diene-2,8-dione	2.81 ± 0.24	**2.97** ± 0.25	2.17 ± 0.15	1.89 ± 0.13	2.08 ± 0.20	1.90 ± 0.15	1.51 ± 0.12	1.48 ± 0.09

RT—retention time; LUHS122—*L. plantarum* strain LUHS122; LUHS210—*L. casei* strain LUHS210; nd—not detectable.

**Table 14:** Characteristics of nutraceutical chewing candies.

Parameters	NCC-1	NCC-2	NCC-3	NCC-4	NCC-5	NCC-6
OA	9.03 ± 0.72	8.09 ± 0.63	9.51 ± 0.76	10.0 ± 0.69	8.52 ± 0.63	9.08 ± 0.82
	**Emotions induced for consumers (from 0 to 1)**					
Neutral	0.003 ± 0.001	0.002 ± 0.001	0.003 ± 0.001	0.002 ± 0.001	0.005 ± 0.002	0.001 ± 0.001
Happy	0.813 ± 0.062	0.534 ± 0.035	0.905 ± 0.057	0.916 ± 0.078	0.734 ± 0.061	0.904 ± 0.048
Sad	0.0001 ± 0.0001	0.0001 ± 0.0001	0.0001 ± 0.0001	0.0001 ± 0.0001	0.0002 ± 0.0001	0.0001 ± 0.0001
Angry	0.0011 ± 0.0002	0.0019 ± 0.0003	0.0003 ± 0.0001	0.0004 ± 0.0001	0.0024 ± 0.0002	0.0005 ± 0.0001
Surprised	0.0240 ± 0.0036	0.0258 ± 0.0025	0.0252 ± 0.0018	0.0312 ± 0.0025	0.0235 ± 0.0019	0.0451 ± 0.0029
Scared	0.0002 ± 0.0001	0.0001 ± 0.0001	0.0001 ± 0.0001	0.0002 ± 0.0001	0.0002 ± 0.0001	0.0001 ± 0.0001
Disgusted	0.0032 ± 0.0003	0.0045 ± 0.0002	0.0024 ± 0.0003	0.0032 ± 0.0002	0.0036 ± 0.0003	0.0027 ± 0.0003
Contempt	0.0020 ± 0.0001	0.0019 ± 0.0002	0.0020 ± 0.0001	0.0020 ± 0.0003	0.0020 ± 0.0002	0.0019 ± 0.0001
Valence	0.250 ± 0.015	0.1404 ± 0.0096	0.3275 ± 0.0120	0.2355 ± 0.0256	0.1581 ± 0.0110	0.1390 ± 0.0098
	**Colour coordinates, NBS**					
L*	50.7 ± 0.1	60.5 ± 0.1	62.0 ± 0.1	63.2 ± 0.1	63.5 ± 0.1	61.5 ± 0.2
a*	3.23 ± 0.15	3.80 ± 0.12	0.550 ± 0.009	0.170 ± 0.006	−0.500 ± 0.009	−0.820 ± 0.007
b*	34.8 ± 0.2	34.9 ± 0.1	30.2 ± 0.2	30.2 ± 0.1	25.1 ± 0.1	24.9 ± 0.2
	**Texture, mJ**					
	3.20 ± 0.18	2.00 ± 0.11	4.00 ± 0.07	2.70 ± 0.10	4.00 ± 0.03	4.10 ± 0.08
	**Images of the NCC**					
	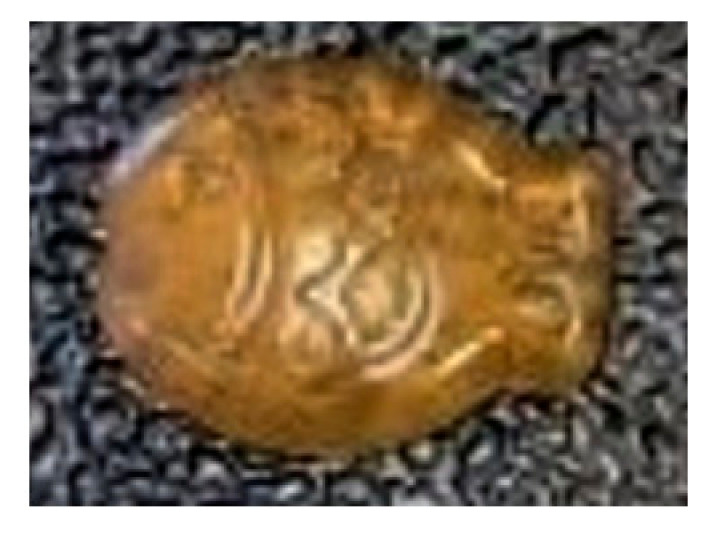	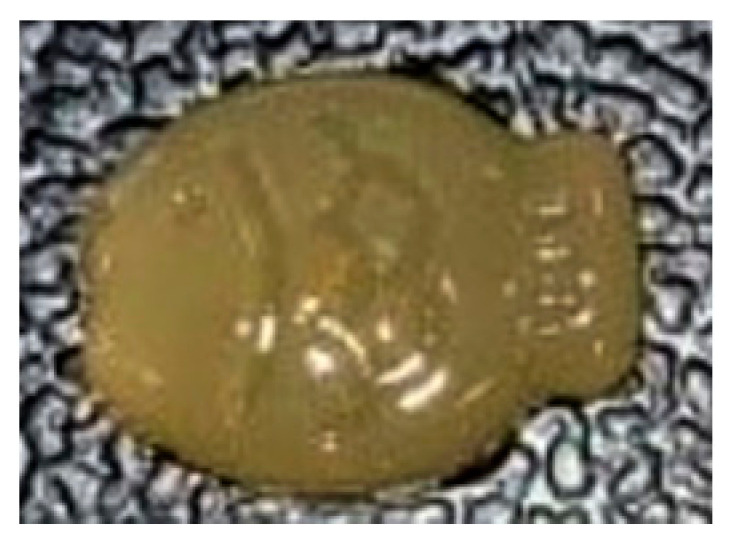	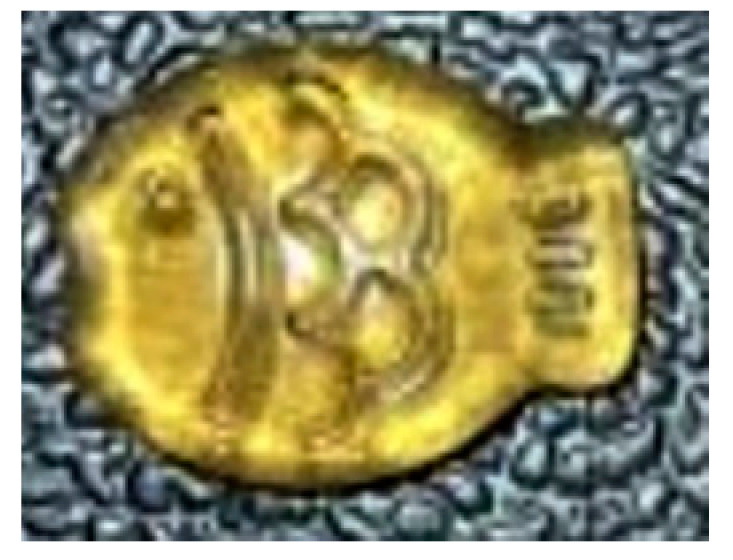	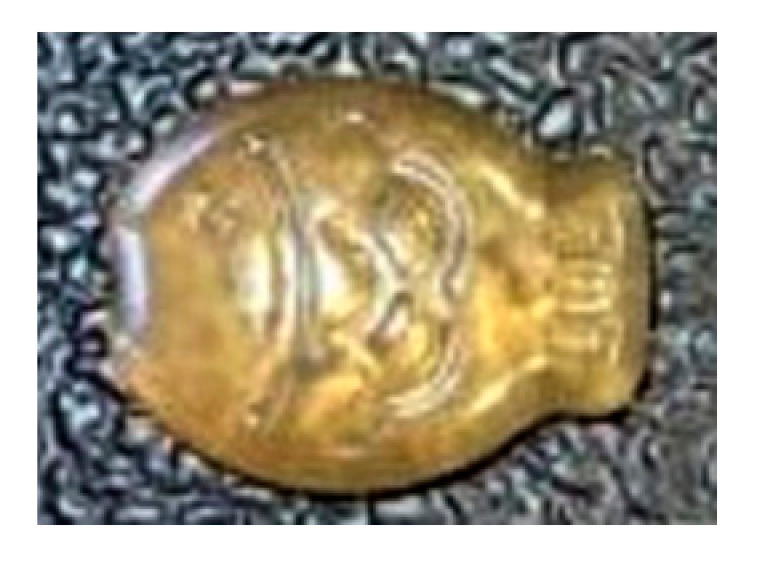	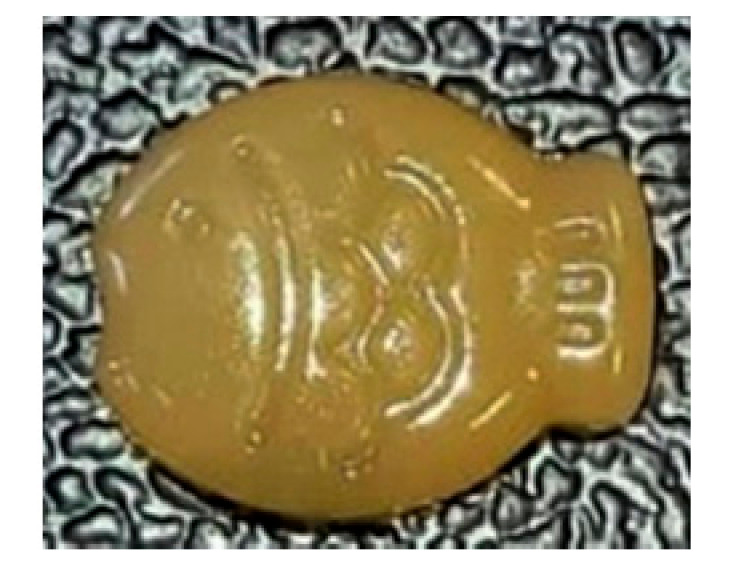	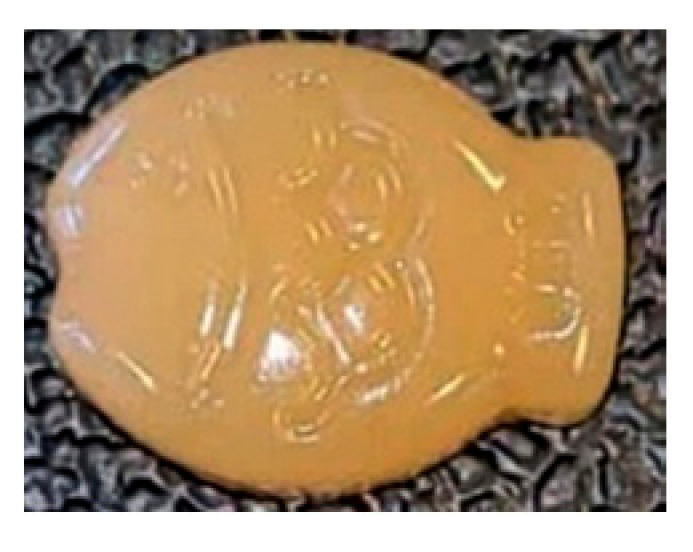

NCC—nutraceutical chewing candies; OA—overall acceptability; L* lightness; a* redness or −a* greenness; b* yellowness or −b* blueness; NBS—National Bureau of Standards units.

## Data Availability

The data are available from the corresponding author upon reasonable request.

## Supplementary Materials

The following are available online at https://www.mdpi.com/article/10.3390/foods10102329/s1, Table S1: Volatile compound profile of non-fermented frozen apple juice, Table S2: Volatile compound profile of cider, Table S3: Volatile compound profile of fermented apple juice.
