# Supplementary material for: Nutraceutical Chewing Candy Formulations Based on Acetic, Alcoholic, and Lactofermented Apple Juice Products
Source: Foods. 2021 Sep 30;10(10):2329. doi: 10.3390/foods10102329 (PMC8535157; doi:10.3390/foods10102329)
Supplement: Supplementary file 1 [file foods-10-02329-s001.zip › foods-1386272-supplementary.pdf]

Supplementary file 1. Volatile compounds profile.

Table S1. Nonfermented frozen apple juice volatile compounds profile.

RT, min	Volatile compound	Nonfer-mented frozen apple juice
2.471	Ethyl Acetate	0.000
3.05	1-Butanol	3.307
4.373	1-Butanol, 3-methyl-	0.000
5.861	Butyrate <ethyl->	0.983
6.159	Propanoic acid, 2-hydroxy-, ethyl ester, (S)-	0.000
6.21	Acetic acid, butyl ester	10.916
7.594	1-Hexanol	30.661
10.245	Formate <heptyl->	0.418
10.492	Hexanoic acid	0.523
10.724	5-Hepten-2-one, 6-methyl-	0.194
10.882	Hept-5-en-2-ol <6-methyl->	0.465
10.947	Butanoate <butyl->	3.637
11.022	Hexanoic acid, ethyl ester	0.000
11.402	Acetic acid, hexyl ester	18.652
11.775	1-Hexanol, 2-ethyl-	0.407
11.896	Benzyl alcohol	0.000
12.1	Butyl 2-methylbutanoate	0.400
12.175	Benzeneacetaldehyde	0.000
12.28	3-Octen-1-ol	0.039
12.514	Ethyl dl-2-hydroxycaproate	0.000
12.531	3,3-Dimethyl-2-pentanol	0.000
12.541	Butanoate, <2-Methylbutyl ->	0.000
12.595	Bicyclo[2.2.1]heptane, 2-methyl-	0.069

12.803	1-Octanol	4.612
13.318	Linalool oxide <cis->	0.098
13.504	Heptanoic acid, ethyl ester	0.116
13.538	1,6-Octadien-3-ol, 3,7-dimethyl-	0.178
13.65	cis-1,2-Cyclohexanedimethanol	0.810
13.817	Acetic acid, nonyl ester	0.279
13.911	Phenethyl alcohol	0.000
13.955	Hexanoic acid, 2-ethyl-	0.346
14.095	Octanoate <methyl->	0.000
14.235	Butanoic acid, 3-hydroxy-, butyl ester	0.314
14.312	2-Undecene, 4-methyl-	0.183
14.553	(S)-(+)-6-Methyl-1-octanol	0.166
14.666	Butyrate <hexyl->	0.121
14.745	Hexanoate <isobutyl->	0.000
14.944	2-Nonenal, (E)-	0.022
15.071	Acetic acid, phenylmethyl ester	0.077
15.125	Phenol, 4-ethyl-	0.000
15.333	Benzoic acid, ethyl ester	0.000
15.383	Butanedioic acid, diethyl ester	0.000
15.472	Octanoic acid	0.000
15.69	Butanoic acid, hexyl ester	5.304
15.783	Octanoate <ethyl->	0.000
15.999	Decanal	0.027
16.02	2-Hexanol, 3,4-dimethyl-	0.000
16.038	3.beta.-Methyl-trans-hexahydrophthalide	0.014
16.096	Acetic acid, octyl ester	0.798
16.158	4-Octanol, 2,4-dimethyl-	0.000
16.287	3-Pentanone, 2,2,4,4-tetramethyl-	0.020
16.385	Phenoxyethanol	0.000
16.492	Citronellol	0.000
16.66	2-Methylbicyclo[4.3.0]non-1(6)-ene	0.000
16.704	Butanoic acid, 2-methyl-, hexyl ester	1.435

17.049	Chavicol	0.000
17.093	Geraniol	0.000
17.174	1,3-Octanediol	2.869
17.218	Cyclooctene, 3-ethenyl-	1.011
17.338	Nonanoic acid	2.259
17.444	1-Decanol	0.000
17.655	Dodecane, 2,6,10-trimethyl-	0.000
17.668	1-Hexanol, 5-methyl-2-(1-methylethyl)-	0.000
17.674	Methyl undecyl ether	0.000
17.807	2-Pyrazoline, 3-ethyl-1-isopropyl-	1.003
17.933	2-Undecanone	0.000
17.936	Undecan-2-one	0.000
17.94	Nonanoate <ethyl->	0.000
18.011	Indole	0.105
18.077	Methyl nonyl carbinol	0.000
18.161	Formamide, N,N-dibutyl-	0.000
18.335	2,4-Decadien-1-ol, (E,E)-	0.000
18.41	Guaiacol <4-vinyl->	0.000
18.679	Ethanol, 2-(2-butoxyethoxy)-, acetate	0.000
18.74	Hydrocinnamic acid	0.000
18.787	Octanoic acid <4-ethyl->	0.000
18.98	n-PROPYL NONYL ETHER	0.000
19.138	10-Methyl-3,4,5,8,9,10-hexahydrooxecin-2-one	0.261
19.247	2,2,4-Trimethyl-1,3-pentanediol diisobutyrate	0.281
19.322	3-Allyl-6-methoxyphenol	0.000
19.358	n-Decanoic acid	0.000
19.475	Succinic acid, butyl ethyl ester	0.000
19.558	2-Hydroxypentadecyl propanoate	0.000
19.686	Propanoic acid, 2-methyl-, 3-hydroxy-2,4,4-trimethylpentyl ester	0.555
19.849	Butyl caprylate	1.390

19.912	2-Buten-1-one, 1-(2,6,6-trimethyl-1,3-cyclohexadien-1-yl)-, (E)-	0.171
19.959	Nonadien-1-yl acetate, <3,6->	0.115
19.974	Decanoate <ethyl->	0.000
20.068	Tetradecane	0.035
20.194	Methyleugenol	0.096
20.263	Dodecanal	0.033
20.401	2,4,7,9-Tetramethyl-5-decyn-4,7-diol	0.061
20.624	Butanedioic acid, ethyl 3-methylbutyl ester	0.000
20.701	Succinic acid, ethyl 3-pentyl ester	0.000
20.742	1-Hexadecanol, 2-methyl-	0.000
21	Octanoic acid, 3-methylbutyl ester	0.000
21.045	(Z)6,(Z)9-Pentadecadien-1-ol	0.286
21.146	5,9,13-Pentadecatrien-2-one, 6,10,14-trimethyl-, (E,E)-	0.165
21.249	2-Isopropyl-5-methyl-1-heptanol	0.191
21.263	Undecanoic acid	0.000
21.526	Dodecanol	0.000
21.576	2,5-Cyclohexadiene-1,4-dione, 2,6-bis(1,1-dimethylethyl)-	0.841
21.615	1-Heptanol, 2,4-diethyl-	0.000
21.682	Oxalic acid, cyclobutyl octadecyl ester	0.000
22.206	Farnesene <(E,E)-, alpha->	0.309
22.275	Phenol, 2,4-bis(1,1-dimethylethyl)-	0.543
22.486	Dichloroacetic acid, 4-hexadecyl ester	0.000
22.486	n-Tridecan-1-ol	0.000
22.564	Hexadecane, 1,16-dichloro-	0.000
22.569	1-Decanol, 2-methyl-	0.000
22.807	Octatriacontane <n->	0.000
23.08	Dodecanoic acid	0.000
23.242	1,6,10-Dodecatrien-3-ol, 3,7,11-trimethyl-, (E)-	0.010
23.546	Hexamethylene diacrylate	0.178
23.704	Dodecanoate <ethyl->	0.000

23.81	Hexadecane	0.044
23.871	Propanoic acid, 2-methyl-, 1-(1,1-dimethylethyl)-2-methyl-1,3-propanediyl ester	0.303
24.083	Tetradecanal	0.000
24.175	Tricyclo[4.4.0.0(2,7)]dec-8-ene-3-methanol, .alpha.,.alpha.,6,8-tetramethyl-, stereoisomer	0.030
24.285	Dodecanoic acid, 1-methylethyl ester	0.000
24.608	Pentadecanoic acid, 3-methylbutyl ester	0.000
24.87	Dihydrojasmonate <methyl-, cis->	0.000
25.447	6,10-Dodecadien-1-ol, 3,7,11-trimethyl-, (E)-(.+/-.)-	0.000
25.615	Methanone, (1-hydroxycyclohexyl)phenyl-	0.026
25.992	trans-Geranylgeraniol	0.000
26.62	Dibutyl adipate	0.013
26.822	3,5-di-tert-Butyl-4-hydroxybenzaldehyde	0.032
27.068	Tetradecanoate <ethyl->	0.000
27.313	2-Propanol, 1-chloro-, phosphate (3:1)	0.022
28.348	1,2-Benzenedicarboxylic acid, bis(2-methylpropyl) ester	0.166
29.199	7,9-Di-tert-butyl-1-oxaspiro(4,5)deca-6,9-diene-2,8-dione	0.921
29.743	Dibutyl phthalate	0.086
30.066	Palmitate <ethyl->	0.000
RT – retention time.		

Table S2. Cider samples volatile compounds profile.

RT, min	Volatile compound	C No.1	C No.2	C No.3	C No.4
2.471	Ethyl Acetate	0.000	0.000	0.000	0.000
3.05	1-Butanol	0.000	0.000	0.000	0.000

4.373	1-Butanol, 3-methyl-	27.939	22.967	29.107	18.910
5.861	Butyrate <ethyl->	0.000	0.000	0.000	0.000
6.159	Propanoic acid, 2-hydroxy-, ethyl ester, (S)-	0.000	2.064	0.000	1.321
6.21	Acetic acid, butyl ester	0.000	0.000	0.000	0.000
7.594	1-Hexanol	6.409	5.969	9.218	5.272
10.245	Formate <heptyl->	0.157	0.074	0.092	0.075
10.492	Hexanoic acid	1.449	1.581	1.661	0.000
10.724	5-Hepten-2-one, 6-methyl-	0.000	0.000	0.000	0.000
10.882	Hept-5-en-2-ol <6-methyl->	0.158	0.157	0.117	0.233
10.947	Butanoate <butyl->	0.000	0.000	0.000	0.000
11.022	Hexanoic acid, ethyl ester	1.996	4.254	3.856	3.777
11.402	Acetic acid, hexyl ester	0.110	0.371	0.267	0.139
11.775	1-Hexanol, 2-ethyl-	0.231	0.191	0.359	0.230
11.896	Benzyl alcohol	0.094	0.048	0.000	0.000
12.1	Butyl 2-methylbutanoate	0.000	0.059	0.000	0.081
12.175	Benzeneacetaldehyde	0.000	0.000	0.000	0.000
12.28	3-Octen-1-ol	0.014	0.000	0.000	0.000
12.514	Ethyl dl-2-hydroxycaproate	0.186	0.047	0.042	0.046
12.531	3,3-Dimethyl-2-pentanol	0.000	0.000	0.000	0.000
12.541	Butanoate, <2-Methylbutyl ->	0.000	0.000	0.000	0.000
12.595	Bicyclo[2.2.1]heptane, 2-methyl-	0.000	0.000	0.000	0.000
12.803	1-Octanol	2.127	1.269	0.000	1.085
13.318	Linalool oxide <cis->	0.110	0.000	0.000	0.000
13.504	Heptanoic acid, ethyl ester	0.000	0.000	0.000	0.000
13.538	1,6-Octadien-3-ol, 3,7-dimethyl-	0.000	0.107	0.122	0.103
13.65	cis-1,2-Cyclohexanedimethanol	0.406	0.224	0.263	0.182
13.817	Acetic acid, nonyl ester	0.000	0.000	0.000	0.000
13.911	Phenethyl alcohol	11.596	7.929	9.996	8.472
13.955	Hexanoic acid, 2-ethyl-	0.000	0.000	0.000	0.000
14.095	Octanoate <methyl->	0.000	0.006	0.000	0.006
14.235	Butanoic acid, 3-hydroxy-, butyl ester	0.102	0.062	0.119	0.088

14.312	2-Undecene, 4-methyl-	0.164	0.111	0.184	0.138
14.553	(S)-(+)-6-Methyl-1-octanol	0.116	0.080	0.127	0.106
14.666	Butyrate <hexyl->	0.000	0.000	0.000	0.000
14.745	Hexanoate <isobutyl->	0.000	0.000	0.000	0.000
14.944	2-Nonenal, (E)-	0.000	0.000	0.000	0.000
15.071	Acetic acid, phenylmethyl ester	0.000	0.000	0.000	0.000
15.125	Phenol, 4-ethyl-	0.000	0.000	0.000	0.000
15.333	Benzoic acid, ethyl ester	0.008	0.000	0.000	0.000
15.383	Butanedioic acid, diethyl ester	8.758	4.782	0.000	4.784
15.472	Octanoic acid	0.000	7.109	4.036	0.000
15.69	Butanoic acid, hexyl ester	0.101	0.000	0.000	12.521
15.783	Octanoate <ethyl->	1.781	8.036	6.767	5.890
15.999	Decanal	0.117	0.177	0.103	0.136
16.02	2-Hexanol, 3,4-dimethyl-	0.000	0.000	0.000	0.000
16.038	3.beta.-Methyl-trans-hexahydrophthalide	0.000	0.000	0.000	0.000
16.096	Acetic acid, octyl ester	0.000	0.000	0.000	0.000
16.158	4-Octanol, 2,4-dimethyl-	1.407	0.000	0.000	0.000
16.287	3-Pentanone, 2,2,4,4-tetramethyl-	0.000	0.023	0.000	0.033
16.385	Phenoxyethanol	0.000	0.000	0.000	0.000
16.492	Citronellol	0.436	0.104	0.179	0.000
16.66	2-Methylbicyclo[4.3.0]non-1(6)-ene	0.000	0.000	0.000	0.000
16.704	Butanoic acid, 2-methyl-, hexyl ester	0.000	0.020	0.027	0.000
17.049	Chavicol	0.577	0.373	0.344	0.664
17.093	Geraniol	0.000	0.000	0.000	0.000
17.174	1,3-Octanediol	3.456	0.780	1.472	0.541
17.218	Cyclooctene, 3-ethenyl-	0.000	0.000	0.000	0.000
17.338	Nonanoic acid	4.766	2.072	6.467	5.404
17.444	1-Decanol	0.000	2.127	0.000	0.000
17.655	Dodecane, 2,6,10-trimethyl-	0.000	0.000	0.000	0.000
17.668	1-Hexanol, 5-methyl-2-(1-methylethyl)-	0.000	0.000	0.000	0.000
17.674	Methyl undecyl ether	0.000	0.000	0.000	0.000
17.807	2-Pyrazoline, 3-ethyl-1-isopropyl-	0.000	0.544	0.797	0.637

17.933	2-Undecanone	0.000	0.000	0.000	0.000
17.936	Undecan-2-one	0.000	0.000	0.000	0.000
17.94	Nonanoate <ethyl->	0.113	0.150	0.149	0.154
18.011	Indole	0.167	0.114	0.137	0.084
18.077	Methyl nonyl carbinol	0.000	0.000	0.000	0.000
18.161	Formamide, N,N-dibutyl-	0.000	0.000	0.000	0.098
18.335	2,4-Decadien-1-ol, (E,E)-	0.000	0.000	0.000	0.000
18.41	Guaiacol <4-vinyl->	0.034	0.020	0.025	0.000
18.679	Ethanol, 2-(2-butoxyethoxy)-, acetate	0.000	0.000	0.000	0.000
18.74	Hydrocinnamic acid	0.000	0.000	0.000	0.000
18.787	Octanoic acid <4-ethyl->	0.149	0.088	0.000	0.111
18.98	n-PROPYL NONYL ETHER	0.127	0.022	0.000	0.000
19.138	10-Methyl-3,4,5,8,9,10-hexahydrooxecin-2-one	0.000	0.029	0.041	0.037
19.247	2,2,4-Trimethyl-1,3-pentanediol diisobutyrate	0.153	0.114	0.196	0.147
19.322	3-Allyl-6-methoxyphenol	3.302	0.630	0.000	0.376
19.358	n-Decanoic acid	1.960	6.908	7.451	11.303
19.475	Succinic acid, butyl ethyl ester	0.000	0.000	0.000	0.000
19.558	2-Hydroxypentadecyl propanoate	0.574	0.000	0.000	0.000
19.686	Propanoic acid, 2-methyl-, 3-hydroxy-2,4,4-trimethylpentyl ester	0.270	0.248	0.333	0.328
19.849	Butyl caprylate	0.000	0.000	0.000	0.000
19.912	2-Buten-1-one, 1-(2,6,6-trimethyl-1,3-cyclohexadien-1-yl)-, (E)-	0.168	0.119	0.156	0.124
19.959	Nonadien-1-yl acetate, <3,6->	0.000	0.000	0.000	0.000
19.974	Decanoate <ethyl->	0.622	6.162	2.862	3.652
20.068	Tetradecane	0.064	0.042	0.043	0.047
20.194	Methyleugenol	0.322	0.053	0.000	0.000
20.263	Dodecanal	0.171	0.115	0.140	0.070
20.401	2,4,7,9-Tetramethyl-5-decyn-4,7-diol	0.000	0.000	0.000	0.000

20.624	Butanedioic acid, ethyl 3-methylbutyl ester	0.630	0.297	0.000	0.275
20.701	Succinic acid, ethyl 3-pentyl ester	0.000	0.000	0.000	0.058
20.742	1-Hexadecanol, 2-methyl-	0.000	0.117	0.000	0.000
21	Octanoic acid, 3-methylbutyl ester	0.000	0.097	0.093	0.000
21.045	(Z)6,(Z)9-Pentadecadien-1-ol	0.000	0.000	0.000	0.000
21.146	5,9,13-Pentadecatrien-2-one, 6,10,14-trimethyl-, (E,E)-	0.308	0.120	0.000	0.087
21.249	2-Isopropyl-5-methyl-1-heptanol	0.000	0.000	0.000	0.000
21.263	Undecanoic acid	0.532	0.323	0.313	0.350
21.526	Dodecanol	0.000	0.000	0.000	0.000
21.576	2,5-Cyclohexadiene-1,4-dione, 2,6-bis(1,1-dimethylethyl)-	2.286	1.427	1.523	1.454
21.615	1-Heptanol, 2,4-diethyl-	0.000	0.000	0.157	0.188
21.682	Oxalic acid, cyclobutyl octadecyl ester	0.484	0.263	0.000	0.344
22.206	Farnesene <(E,E)-, alpha->	0.000	0.000	0.049	0.000
22.275	Phenol, 2,4-bis(1,1-dimethylethyl)-	5.651	3.521	2.495	3.777
22.486	Dichloroacetic acid, 4-hexadecyl ester	0.138	0.000	0.000	0.000
22.486	n-Tridecan-1-ol	0.000	0.054	0.000	0.000
22.564	Hexadecane, 1,16-dichloro-	0.000	0.000	0.000	0.000
22.569	1-Decanol, 2-methyl-	0.000	0.000	0.000	0.151
22.807	Octatriacontane <n->	0.000	0.000	0.000	0.000
23.08	Dodecanoic acid	0.282	0.430	0.648	0.493
23.242	1,6,10-Dodecatrien-3-ol, 3,7,11-trimethyl-, (E)-	0.383	0.122	0.100	0.073
23.546	Hexamethylene diacrylate	0.227	0.127	0.211	0.190
23.704	Dodecanoate <ethyl->	0.113	0.158	0.341	0.303
23.81	Hexadecane	0.067	0.044	0.000	0.054
23.871	Propanoic acid, 2-methyl-, 1-(1,1-dimethylethyl)-2-methyl-1,3-propanediyl ester	0.781	0.522	0.679	0.523
24.083	Tetradecanal	0.000	0.000	0.000	0.000

24.175	Tricyclo[4.4.0.0(2,7)]dec-8-ene-3-methanol, .alpha.,.alpha.,6,8-tetramethyl-, stereoisomer	0.043	0.000	0.000	0.000
24.285	Dodecanoic acid, 1-methylethyl ester	0.000	0.000	0.024	0.025
24.608	Pentadecanoic acid, 3-methylbutyl ester	0.000	0.000	0.000	0.000
24.87	Dihydrojasmonate <methyl-, cis->	0.000	0.000	0.000	0.000
25.447	6,10-Dodecadien-1-ol, 3,7,11-trimethyl-, (E)-(.+/-.)-	0.083	0.000	0.082	0.000
25.615	Methanone, (1-hydroxycyclohexyl)phenyl-	0.000	0.000	0.000	0.041
25.992	trans-Geranylgeraniol	0.000	0.076	0.135	0.000
26.62	Dibutyl adipate	0.054	0.032	0.048	0.039
26.822	3,5-di-tert-Butyl-4-hydroxybenzaldehyde	0.175	0.112	0.149	0.125
27.068	Tetradecanoate <ethyl->	0.046	0.049	0.068	0.061
27.313	2-Propanol, 1-chloro-, phosphate (3:1)	0.070	0.039	0.053	0.029
28.348	1,2-Benzenedicarboxylic acid, bis(2-methylpropyl) ester	0.784	0.686	0.944	0.657
29.199	7,9-Di-tert-butyl-1-oxaspiro(4,5)deca-6,9-diene-2,8-dione	3.408	2.337	3.745	2.584
29.743	Dibutyl phthalate	0.457	0.481	0.802	0.526
30.066	Palmitate <ethyl->	0.022	0.035	0.084	0.074
RT – retention time; C – cider samples.					

Table S3. Fermented apple juice volatile compounds profile.

RT, min	Volatile compound	Frozen apple juice				Fresh apple juice			
		Lactic acid bacteria strains used for fermentation							
		LUHS 122		LUHS210		LUHS 122		LUHS 210	
		Duration of fermentation, hours							
		24	**48**	24	48	24	48	24	48
2.471	Ethyl Acetate	0.000	**0.000**	0.000	0.000	0.000	2.834	2.388	0.000
3.05	1-Butanol	3.578	**4.496**	5.591	4.992	1.300	1.473	1.565	1.902

4.373	1-Butanol, 3-methyl-	0.884	**0.620**	2.361	1.867	1.728	4.065	8.333	10.256
5.861	Butyrate <ethyl->	1.035	**0.811**	0.000	0.000	0.816	1.059	0.000	0.000
6.159	Propanoic acid, 2-hydroxy-, ethyl ester, (S)-	0.000	**0.000**	0.000	0.000	0.000	0.000	0.000	0.000
6.21	Acetic acid, butyl ester	11.664	**10.620**	1.796	1.778	9.171	1.966	0.991	1.102
7.594	1-Hexanol	36.389	**40.461**	49.134	44.828	33.086	38.704	36.939	40.181
10.245	Formate <heptyl->	0.505	**0.501**	0.643	0.622	0.505	0.495	0.360	0.412
10.492	Hexanoic acid	0.913	**1.070**	1.245	1.109	1.046	1.479	1.441	1.672
10.724	5-Hepten-2-one, 6-methyl-	0.240	**0.270**	0.404	0.399	0.280	0.313	0.331	0.393
10.882	Hept-5-en-2-ol <6-methyl->	0.536	**0.599**	1.017	0.902	0.600	0.648	0.841	1.513
10.947	Butanoate <butyl->	2.476	**2.204**	0.903	0.989	2.523	1.444	0.514	0.000
11.022	Hexanoic acid, ethyl ester	0.000	**0.000**	0.000	0.000	0.000	0.000	1.454	1.689
11.402	Acetic acid, hexyl ester	8.414	**5.536**	3.175	2.984	6.376	3.944	2.715	3.071
11.775	1-Hexanol, 2-ethyl-	0.663	**0.599**	0.871	9.407	8.146	8.880	8.132	8.867
11.896	Benzyl alcohol	0.189	**0.166**	0.000	0.000	0.000	0.000	0.000	0.000
12.1	Butyl 2-methylbutanoate	0.000	**0.000**	0.000	0.000	0.184	0.168	0.000	0.130
12.175	Benzeneacetaldehyde	0.409	**0.311**	0.526	0.497	0.000	0.000	0.000	0.000
12.28	3-Octen-1-ol	0.085	**0.077**	0.078	0.072	0.063	0.000	0.000	0.000
12.514	Ethyl dl-2-hydroxycaproate	0.000	**0.092**	0.000	0.000	0.000	0.038	0.000	0.000
12.531	3,3-Dimethyl-2-pentanol	0.000	**0.000**	0.000	0.153	0.000	0.000	0.117	0.166
12.541	Butanoate, <2-Methylbutyl ->	0.000	**0.000**	0.000	0.000	0.000	0.000	0.000	0.000
12.595	Bicyclo[2.2.1]heptane, 2-methyl-	0.039	**0.077**	0.062	0.091	0.034	0.000	0.000	0.000
12.803	1-Octanol	4.299	**4.065**	3.979	4.092	3.236	2.670	1.234	1.400
13.318	Linalool oxide <cis->	0.318	**0.194**	0.300	0.305	0.523	0.217	0.282	0.277
13.504	Heptanoic acid, ethyl ester	0.000	**0.000**	0.000	0.000	0.000	0.000	0.166	0.224
13.538	1,6-Octadien-3-ol, 3,7-dimethyl-	0.200	**0.182**	0.281	0.257	0.000	0.000	0.311	0.322
13.65	cis-1,2-Cyclohexanedimethanol	0.726	**0.709**	0.951	0.902	0.930	0.902	0.881	1.104
13.817	Acetic acid, nonyl ester	0.000	**0.000**	0.000	0.000	0.000	0.000	0.000	0.000
13.911	Phenethyl alcohol	0.661	**0.835**	0.963	0.496	1.411	2.617	3.481	3.814
13.955	Hexanoic acid, 2-ethyl-	0.709	**0.512**	0.715	0.554	0.611	0.000	0.000	0.000
14.095	Octanoate <methyl->	0.000	**0.000**	0.000	0.000	0.036	0.047	0.000	0.000
14.235	Butanoic acid, 3-hydroxy-, butyl ester	0.358	**0.354**	0.399	0.388	0.362	0.380	0.354	0.394
14.312	2-Undecene, 4-methyl-	0.279	**0.232**	0.286	0.284	0.240	0.275	0.315	0.326

14.553	(S)-(+)-6-Methyl-1-octanol	0.197	**0.184**	0.209	0.221	0.169	0.189	0.260	0.287
14.666	Butyrate <hexyl->	0.000	**0.000**	0.000	0.000	0.000	0.000	0.000	0.000
14.745	Hexanoate <isobutyl->	0.000	**0.000**	0.000	0.000	0.000	0.000	0.065	0.096
14.944	2-Nonenal, (E)-	0.032	**0.000**	0.000	0.048	0.000	0.000	0.030	0.051
15.071	Acetic acid, phenylmethyl ester	0.000	**0.000**	0.000	0.000	0.000	0.000	0.000	0.000
15.125	Phenol, 4-ethyl-	0.000	**0.000**	0.000	0.000	0.388	0.000	0.000	0.000
15.333	Benzoic acid, ethyl ester	0.000	**0.000**	0.000	0.000	0.000	0.000	0.000	0.000
15.383	Butanedioic acid, diethyl ester	0.000	**0.000**	0.000	0.000	0.000	0.000	0.000	0.000
15.472	Octanoic acid	0.000	**0.000**	0.000	0.000	0.000	0.000	0.000	0.000
15.69	Butanoic acid, hexyl ester	0.626	**0.666**	0.411	0.523	0.425	0.448	0.431	0.506
15.783	Octanoate <ethyl->	0.116	**0.000**	0.252	0.233	0.267	0.639	2.261	2.660
15.999	Decanal	0.089	**0.000**	0.188	0.000	0.000	0.000	0.000	0.000
16.02	2-Hexanol, 3,4-dimethyl-	0.000	**0.084**	0.000	0.141	0.093	0.000	0.102	0.000
16.038	3.beta.-Methyl-trans-hexahydrophthalide	0.000	**0.000**	0.000	0.000	0.000	0.000	0.000	0.000
16.096	Acetic acid, octyl ester	0.000	**0.000**	0.000	0.000	0.000	0.000	0.000	0.000
16.158	4-Octanol, 2,4-dimethyl-	0.000	**0.000**	0.000	0.000	0.000	0.000	0.000	0.000
16.287	3-Pentanone, 2,2,4,4-tetramethyl-	0.035	**0.026**	0.059	0.000	0.000	0.031	0.023	0.024
16.385	Phenoxyethanol	0.000	**0.000**	0.042	0.000	0.000	0.000	0.000	0.000
16.492	Citronellol	0.000	**0.000**	0.000	0.000	0.000	0.000	0.000	0.000
16.66	2-Methylbicyclo[4.3.0]non-1(6)-ene	0.000	**0.000**	0.000	0.000	0.000	0.000	0.000	0.000
16.704	Butanoic acid, 2-methyl-, hexyl ester	0.189	**0.130**	0.532	0.467	0.108	0.142	0.212	0.227
17.049	Chavicol	0.000	**0.000**	0.000	0.000	0.000	0.000	0.000	0.000
17.093	Geraniol	0.000	**0.000**	0.000	0.000	0.610	0.000	0.202	0.000
17.174	1,3-Octanediol	2.630	**3.127**	5.480	5.039	4.331	3.228	4.904	5.798
17.218	Cyclooctene, 3-ethenyl-	0.952	**0.737**	0.000	0.000	0.000	0.867	0.000	0.000
17.338	Nonanoic acid	4.478	**5.529**	5.198	5.365	4.486	6.319	5.518	2.191
17.444	1-Decanol	0.000	**0.000**	0.000	0.000	0.000	0.000	0.000	0.399
17.655	Dodecane, 2,6,10-trimethyl-	0.000	**0.079**	0.100	0.000	0.000	0.000	0.000	0.000
17.668	1-Hexanol, 5-methyl-2-(1-methylethyl)-	0.000	**0.000**	0.000	0.000	0.000	0.105	0.000	0.063
17.674	Methyl undecyl ether	0.000	**0.000**	0.000	0.000	0.139	0.000	0.133	0.000
17.807	2-Pyrazoline, 3-ethyl-1-isopropyl-	1.447	**1.304**	1.620	0.000	0.000	0.000	0.000	0.000
17.933	2-Undecanone	0.303	**0.000**	0.000	0.000	0.330	0.000	0.000	0.000

17.936	Undecan-2-one	0.000	**0.000**	0.132	0.000	0.000	0.000	0.000	0.000
17.94	Nonanoate <ethyl->	0.000	**0.000**	0.000	0.000	0.000	0.143	0.460	0.324
18.011	Indole	0.110	**0.111**	0.110	0.111	0.090	0.099	0.083	0.064
18.077	Methyl nonyl carbinol	0.000	**0.000**	0.000	0.000	0.066	0.000	0.000	0.000
18.161	Formamide, N,N-dibutyl-	0.000	**0.000**	0.000	0.165	0.111	0.102	0.102	0.109
18.335	2,4-Decadien-1-ol, (E,E)-	0.000	**0.000**	0.000	0.029	0.000	0.000	0.000	0.000
18.41	Guaiacol <4-vinyl->	0.000	**0.000**	0.000	0.000	0.000	0.000	0.000	0.000
18.679	Ethanol, 2-(2-butoxyethoxy)-, acetate	0.000	**0.000**	0.000	0.000	0.000	0.000	0.295	0.000
18.74	Hydrocinnamic acid	0.000	**0.000**	0.000	0.000	0.000	0.000	0.000	0.000
18.787	Octanoic acid <4-ethyl->	0.000	**0.000**	0.000	0.000	0.000	0.000	0.000	0.000
18.98	n-PROPYL NONYL ETHER	0.183	**0.180**	0.000	0.000	0.134	0.000	0.000	0.000
19.138	10-Methyl-3,4,5,8,9,10-hexahydrooxecin-2-one	0.199	**0.227**	0.164	0.165	0.205	0.244	0.173	0.187
19.247	2,2,4-Trimethyl-1,3-pentanediol diisobutyrate	0.328	**0.325**	0.360	0.284	0.267	0.285	0.353	0.355
19.322	3-Allyl-6-methoxyphenol	1.913	**0.000**	0.000	0.000	3.495	2.118	3.340	0.000
19.358	n-Decanoic acid	0.000	**1.664**	0.000	1.024	0.000	0.000	0.000	0.000
19.475	Succinic acid, butyl ethyl ester	0.000	**0.000**	0.000	0.000	0.000	0.000	0.000	0.000
19.558	2-Hydroxypentadecyl propanoate	0.000	**0.000**	0.000	0.000	0.000	0.000	0.000	0.000
19.686	Propanoic acid, 2-methyl-, 3-hydroxy-2,4,4-trimethylpentyl ester	0.540	**0.492**	0.571	0.472	0.451	0.440	0.620	0.626
19.849	Butyl caprylate	0.307	**0.162**	0.000	0.000	0.000	0.000	0.000	0.000
19.912	2-Buten-1-one, 1-(2,6,6-trimethyl-1,3-cyclohexadien-1-yl)-, (E)-	0.085	**0.109**	0.158	0.168	0.250	0.153	0.164	0.205
19.959	Nonadien-1-yl acetate, <3,6->	0.000	**0.000**	0.000	0.000	0.000	0.000	0.000	0.000
19.974	Decanoate <ethyl->	0.000	**0.068**	0.000	0.000	0.000	0.124	0.177	0.220
20.068	Tetradecane	0.074	**0.058**	0.056	0.061	0.091	0.113	0.082	0.092
20.194	Methyleugenol	0.095	**0.090**	0.081	0.105	0.074	0.084	0.083	0.120
20.263	Dodecanal	0.078	**0.073**	0.076	0.039	0.052	0.066	0.054	0.050
20.401	2,4,7,9-Tetramethyl-5-decyn-4,7-diol	0.067	**0.077**	0.000	0.000	0.000	0.000	0.000	0.000
20.624	Butanedioic acid, ethyl 3-methylbutyl ester	0.000	**0.000**	0.000	0.000	0.000	0.000	0.000	0.000
20.701	Succinic acid, ethyl 3-pentyl ester	0.000	**0.000**	0.000	0.000	0.000	0.000	0.000	0.000

20.742	1-Hexadecanol, 2-methyl-	0.000	**0.000**	0.000	0.000	0.000	0.000	0.000	0.000
21	Octanoic acid, 3-methylbutyl ester	0.000	**0.000**	0.000	0.000	0.000	0.000	0.000	0.000
21.045	(Z)6,(Z)9-Pentadecadien-1-ol	0.000	**0.000**	0.000	0.000	0.000	0.000	0.000	0.000
21.146	5,9,13-Pentadecatrien-2-one, 6,10,14-trimethyl-, (E,E)-	0.000	**0.050**	0.068	0.067	0.000	0.062	0.000	0.082
21.249	2-Isopropyl-5-methyl-1-heptanol	0.000	**0.063**	0.299	0.266	0.000	0.364	0.245	0.000
21.263	Undecanoic acid	0.000	**0.000**	0.000	0.000	0.000	0.000	0.000	0.000
21.526	Dodecanol	0.000	**0.000**	0.000	0.311	0.654	0.459	0.000	0.000
21.576	2,5-Cyclohexadiene-1,4-dione, 2,6-bis(1,1-dimethylethyl)-	0.572	**0.657**	0.650	0.581	0.560	0.580	0.850	0.539
21.615	1-Heptanol, 2,4-diethyl-	0.000	**0.000**	0.000	0.000	0.172	0.000	0.000	0.000
21.682	Oxalic acid, cyclobutyl octadecyl ester	0.000	**0.000**	0.256	0.000	0.000	0.000	0.000	0.000
22.206	Farnesene <(E,E)-, alpha->	0.392	**0.247**	0.750	0.512	0.553	0.437	0.320	0.319
22.275	Phenol, 2,4-bis(1,1-dimethylethyl)-	3.155	**2.588**	2.171	1.837	3.867	3.726	2.312	2.189
22.486	Dichloroacetic acid, 4-hexadecyl ester	0.000	**0.000**	0.000	0.000	0.000	0.000	0.000	0.000
22.486	n-Tridecan-1-ol	0.000	**0.000**	0.000	0.000	0.000	0.000	0.000	0.000
22.564	Hexadecane, 1,16-dichloro-	0.000	**0.000**	0.000	0.000	0.000	0.149	0.000	0.000
22.569	1-Decanol, 2-methyl-	0.000	**0.000**	0.000	0.000	0.119	0.000	0.000	0.000
22.807	Octatriacontane <n->	0.000	**0.000**	0.000	0.031	0.000	0.000	0.000	0.000
23.08	Dodecanoic acid	0.098	**0.085**	0.058	0.037	0.166	0.088	0.067	0.032
23.242	1,6,10-Dodecatrien-3-ol, 3,7,11-trimethyl-, (E)-	0.000	**0.000**	0.000	0.000	0.000	0.000	0.000	0.000
23.546	Hexamethylene diacrylate	0.257	**0.249**	0.345	0.287	0.317	0.306	0.307	0.325
23.704	Dodecanoate <ethyl->	0.000	**0.000**	0.000	0.000	0.000	0.000	0.021	0.058
23.81	Hexadecane	0.067	**0.063**	0.000	0.092	0.117	0.101	0.090	0.104
23.871	Propanoic acid, 2-methyl-, 1-(1,1-dimethylethyl)-2-methyl-1,3-propanediyl ester	0.435	**0.361**	0.289	0.250	0.339	0.254	0.201	0.170
24.083	Tetradecanal	0.000	**0.000**	0.019	0.000	0.027	0.000	0.000	0.000
24.175	Tricyclo[4.4.0.0(2,7)]dec-8-ene-3-methanol, .alpha.,.alpha.,6,8-tetramethyl-, stereoisomer	0.000	**0.000**	0.000	0.000	0.000	0.000	0.000	0.000

24.285	Dodecanoic acid, 1-methylethyl ester	0.000	**0.000**	0.000	0.000	0.000	0.000	0.000	0.000
24.608	Pentadecanoic acid, 3-methylbutyl ester	0.000	**0.000**	0.000	0.000	0.000	0.000	0.000	0.062
24.87	Dihydrojasmonate <methyl-, cis->	0.066	**0.000**	0.000	0.033	0.020	0.000	0.033	0.000
25.447	6,10-Dodecadien-1-ol, 3,7,11-trimethyl-, (E)-(.+/-.)-	0.000	**0.000**	0.000	0.000	0.000	0.000	0.000	0.000
25.615	Methanone, (1-hydroxycyclohexyl)phenyl-	0.054	**0.059**	0.068	0.065	0.072	0.070	0.054	0.061
25.992	trans-Geranylgeraniol	0.085	**0.000**	0.084	0.060	0.078	0.063	0.056	0.052
26.62	Dibutyl adipate	0.023	**0.034**	0.023	0.017	0.027	0.000	0.000	0.000
26.822	3,5-di-tert-Butyl-4-hydroxybenzaldehyde	0.068	**0.116**	0.067	0.066	0.000	0.000	0.000	0.000
27.068	Tetradecanoate <ethyl->	0.000	**0.042**	0.000	0.000	0.000	0.000	0.000	0.000
27.313	2-Propanol, 1-chloro-, phosphate (3:1)	0.037	**0.026**	0.048	0.000	0.000	0.000	0.000	0.000
28.348	1,2-Benzenedicarboxylic acid, bis(2-methylpropyl) ester	0.626	**0.651**	0.556	0.445	0.453	0.400	0.283	0.270
29.199	7,9-Di-tert-butyl-1-oxaspiro(4,5)deca-6,9-diene-2,8-dione	2.813	**2.970**	2.167	1.892	2.077	1.899	1.512	1.482
29.743	Dibutyl phthalate	0.569	**0.594**	0.560	0.487	0.511	0.463	0.349	0.350
30.066	Palmitate <ethyl->	0.038	**0.052**	0.042	0.037	0.034	0.025	0.033	0.038

RT – retention time; LUHS122 - *L. plantarum* strain; LUHS210 - *L. casei* strain.
